# Isomeric Ag_14_ Nanoclusters With Distinct Photophysical and Nanomechanical Properties

**DOI:** 10.1002/advs.76657

**Published:** 2026-07-20

**Authors:** Vivek Yadav, Arijit Jana, Maya Khatun, Harshita Nagar, Sergei Lebedkin, Amit Mondal, Swetashree Acharya, Sami Malola, Sudhadevi Antharjanam, Moses Egor, Pijush Ghosh, Tomas Base, Manfred Kappes, Hannu Häkkinen, Thalappil Pradeep

**Affiliations:** ^1^ Department of Chemistry Indian Institute of Technology Madras Chennai India; ^2^ Departments of Physics and Chemistry Nanoscience Center University of Jyväskylä Jyväskylä Finland; ^3^ Institute of Physical Chemistry Karlsruhe Institute of Technology (KIT) Karlsruhe Germany; ^4^ Institute of Nanotechnology Karlsruhe Institute of Technology (KIT) Karlsruhe Germany; ^5^ Department of Applied Mechanics and Biomedical Engineering Indian Institute of Technology Madras Chennai India; ^6^ Sophisticated Analytical Instruments Facility (SAIF) Indian Institute of Technology Madras Chennai India; ^7^ Department of Syntheses Institute of Inorganic Chemistry The Czech Academy of Science Prague Czech Republic; ^8^ Department of Chemistry Busitema University Tororo Uganda

**Keywords:** isomerism, photoluminescence, silver nanoclusters, structure‐property relationships

## Abstract

Atomically precise luminescent nanoclusters (NCs) with positional isomerism originating from the coordinating ligand shell represent an important group of functional nanomaterials. We report the synthesis of two neutral isomeric [Ag_14_(CBDT)_6_(TPP)_4_] NCs (where CBDT‐H_2_ = *ortho*‐carborane‐9,12‐dithiol, TPP = triphenylphosphine) having a stable 2e^−^ superatomic configuration with face‐centered cubic (fcc) Ag_6_@Ag_8_ core–shell geometry, where TPP ligand coordination makes the structural distinction. The positioning of four TPP ligands on the outer four Ag atoms results in distinct distortions of the Ag_8_ cubic shell and yields two Ag_14_ isomeric NCs: Ag_14_T (tetrahedral TPP binding) and Ag_14_S (square‐planar TPP binding). Density functional theory (DFT) calculations confirm a narrowed HOMO‐LUMO gap for Ag_14_S. The isomeric NCs exhibit markedly distinct photoluminescence (PL) properties, with room‐temperature green and red phosphorescence for Ag_14_T and Ag_14_S, respectively. The former NC shows an especially complex PL behavior with multi‐band emission at low temperatures. Furthermore, nanoindentation studies reveal slightly higher stiffness and hardness for Ag_14_S crystals than the other isomer. These findings establish phosphine binding as a versatile strategy to tune structure, luminescence, and mechanical characteristics in atomically precise NCs, enabling tailored platforms for optoelectronics and nanomechanics applications.

## Graphical abstract

Ligand‐shell isomerism in atomically precise Ag_14_ nanoclusters is demonstrated to regulate core distortion, photoluminescence, and mechanical properties. Subtle changes in phosphine ligand arrangement drive a pronounced emission shift from green to red and alter crystal rigidity, revealing a direct structure‐property correlation that offers new design strategies for functional nanomaterials.

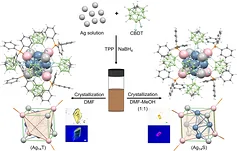

## 1 Introduction

Atomically precise coinage metal NCs have garnered considerable interest because of their versatile structures, quantum confinement properties, adjustable electronic configurations, and intriguing physicochemical properties [1, 2, 3]. These features make them highly promising for use in chemical sensing, biological imaging, and catalytic processes. Their precisely defined molecular architectures make them excellent model systems for exploring the correlations between structure and properties [4, 5, 6, 7, 8, 9]. Successful synthesis of isomeric metal NCs (encompassing chiral, structural, and quasi‐structural isomerism with minimal variations in composition and framework) advances progress in this area of research. Several structural and quasi‐structural isomers of metal NCs have been identified, highlighting metal or ligand based isomerism [10, 11, 12, 13, 14, 15, 16, 17, 18, 19, 20]. Most of the reported studies, however, concentrate on gold‐based and bimetallic systems [13, 14, 15, 16, 17, 18], while only a few involve silver‐based clusters [10, 19, 20]. Moreover, these isomeric silver NCs are typically stabilized by soft ligands such as thiolates and alkynyl groups [10, 11, 12]. NCs possess atomic precision in size, composition, and crystal packing arrangements, which results in properties that depend sensitively on individual metal atoms or ligand molecules [21, 22, 23, 24, 25, 26, 27]. Hence, engineering of the structural aspects of metal NCs is crucial for the fine control of their physical and chemical behaviors.

Significant advancements have been made in tailoring the interfaces of metal nanoclusters using phosphines, thiols, alkynyls, halides, and N‐heterocyclic carbenes ligands [3, 28, 29]. These ligands effectively enhance the surface reactivity of NCs, thereby driving enhancements in properties and catalytic applications [29, 30, 31, 32, 33, 34, 35]. Precise modification of surface sites on metal NCs offers a powerful strategy for tuning both their intrinsic stability and functional attributes. However, changes to the protective ligand environment frequently invoke partial disassembly, extensive reorganization, or even a complete restructuring of the cluster's metallic core [36, 37, 38, 39, 40]. The photoluminescence (PL) of noble metal NCs is closely governed by the cooperative interactions among their structural elements, namely the metallic core–shell, and surface ligands. Modifying the ligands and alloying with other metals have been studied as an effective approach for tuning these interactions and influencing the resulting emission [24, 41, 42, 43, 44, 45]. To the best of our knowledge, the effect of metal core/ligand shell isomerism on structurally resolved Ag NC luminescence, triggered by positional changes of secondary ligands, has not been reported.

Gaining insight into the mechanical properties of NC crystals is essential for their deployment in nanoelectronics, yet investigations in this area remain limited. Nanoindentation has been effectively utilized to characterize the mechanical behavior of Ag_29_ and Ag_40/46_ NC crystals, offering valuable data on parameters such as hardness, elastic modulus, stiffness, and plastic deformation [46, 47, 48]. Such findings play a crucial role in correlating the mechanical performance with the structural attributes of the NCs, thereby aiding in the understanding of their structure‐property relationships [49, 50].

The fcc geometry of Ag_14_ clusters with Ag_6_@Ag_8_ core–shell architecture is well established with extensive evidence on the modulations in the metallic core structure, staple arrangements, and ligand interactions [38, 51, 52, 53, 54]. Although triphenylphosphine (TPP) acts as a secondary ligand, it plays a significant role in shaping the interplay between the metal core and the surrounding staple motifs [52, 53]. Previous reports indicate that the TPP coordinates to the metal atoms present at the corners of the cube, pulling them out from the core and anchoring them at the stable peripheral positions, thereby inducing distortions within the metal core [38, 52, 53]. Despite these observations, a critical knowledge gap persists concerning the precise control over the number and type of spatial arrangements of TPP ligands and the resulting impact on core distortion and geometry, with the emergence of distinct isomeric structures. Bridging this gap is essential for elucidating structure‐property relationships in ligand‐protected isomeric silver NCs.

Herein, we report two isomeric silver NCs, Ag_14_T and Ag_14_S, each composed of 14 Ag atoms, 6 CBDT, and 4 TPP ligands. The systematic positioning of TPP exerts a decisive influence on the geometry of the metal core. For Ag_14_T, four TPPs are attached to the outer corner Ag atoms of the fcc‐Ag_14_ structure at the tetrahedral positions, whereas in Ag_14_S, 4 TPP attached in a square planar manner. The coordination of TPP to surface Ag atoms induces noticeable structural distortions by pulling the bound Ag atoms slightly outward from the core, disrupting the ideal fcc geometry. DFT calculations provided insight into the molecular orbitals governing their characteristic electronic transitions. Variation in the core architectures and solid‐state intermolecular packing collectively modulates their emission behavior, leading to surprisingly distinct green (Ag_14_T) to red (Ag_14_S) luminescence at room temperature. Lifetime and temperature dependent investigations reveal triplet emissive excited states for these compounds. Furthermore, nanomechanical studies reveal that Ag_14_S exhibits greater rigidity compared to Ag_14_T. Overall, this study introduces an example of isomeric silver NCs with the same nuclearity but markedly different optical and mechanical properties, underscoring the crucial role of secondary ligands in tailoring structure‐property relationships.

## 2 Results and Discussion

### 2.1 Synthesis and Crystallization

The synthesis of Ag_14_T and Ag_14_S NCs commenced with the dissolution of silver nitrate (AgNO_3_) in a mixed solvent system of methanol and dichloromethane (MeOH/DCM). Separately, CBDT‐H_2_ was prepared in DCM and subsequently introduced into the AgNO_3_ solution. TPP was then added, and the reaction mixture was stirred vigorously for 15 min. An aqueous NaBH_4_ solution was added dropwise, effecting a gradual color change to yellow. The mixture was stirred for a further 3 h, producing a greenish‐yellow precipitate, which was isolated via centrifugation and washed twice with MeOH. The purified product was dissolved in N,N‐dimethylformamide (DMF). Single crystals of Ag_14_T were obtained via slow evaporation from DMF at room temperature, whereas Ag_14_S crystals were grown under identical conditions from a 1:1 DMF/MeOH mixture (Figure 1 and Figure S1). After 2 weeks in the respective solvents, greenish yellow (Ag_14_T) and orangish yellow (Ag_14_S) crystals suitable for X‐ray diffraction were isolated. Additional details of synthesis are provided in the experimental section. The presence of MeOH is proposed to influence ligand‐shell dynamics and stability of intermediates during crystallization, thereby promoting structural reorganization and enabling the stabilization of the Ag_14_ isomer [55].

**FIGURE 1:**
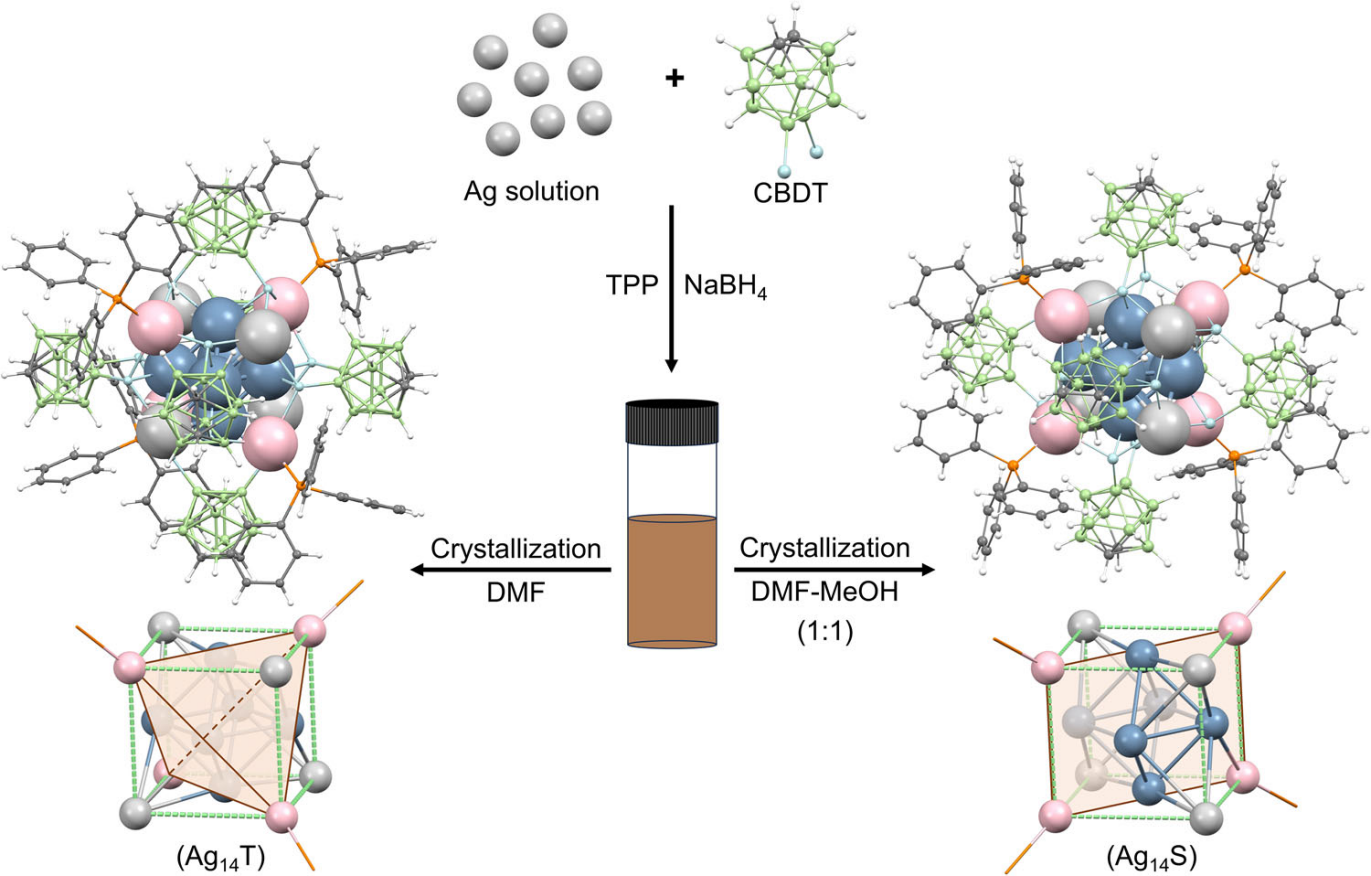
Schematic illustration of the synthesis and solvent‐specific crystallization of Ag_14_T and Ag_14_S NCs. Color code–blue: core silver, metallic gray: core silver, pink: staple silver, orange: phosphorus, cyan: sulfur, green: boron, dark gray: carbon, and white: hydrogen. The tetrahedral and square planar binding of TPP on Ag leads to distinct structures, which are illustrated by the models below.

### 2.2 SC‐XRD Structural Analysis of Ag_14_T and Ag_14_S

Single‐crystal X‐ray diffraction (SC‐XRD) analysis revealed that both Ag_14_T and Ag_14_S isomers share an identical molecular composition, [Ag_14_(CBDT)_6_(TPP)_4_], consisting of 14 silver atoms coordinated by six CBDT and four TPP ligands. Although the overall structure looks like the distorted fcc‐Ag_14_ structure with the metallic core as an Ag_6_ octahedron encapsulated by a cubic Ag_8_ shell. The coordination preferences of TPP specifically tune the outer cubic Ag_8_ shell, which decides structure‐specific luminescence and nanomechanical properties in crystalline state of these isomers. In Ag_14_T, the four TPP ligands coordinate to the outer silver atoms in a tetrahedral fashion, whereas in Ag_14_S, the same number of TPP ligands adopt a square‐planar configuration (Figures 1 and 2g,h). It is known that when TPP binds with the outer metal atoms of certain NCs, it will pull the bonded metal atom out of the NC core, inducing local distortions in the outer metal shell [52, 53]. This phenomenon is also evident in the present systems, where the core geometry is modulated by the positioning of the TPP ligands. Ag_14_T crystallizes in the triclinic crystal system (space group *P* $\bar{1}$), while Ag_14_S adopts a monoclinic system (space group *P*2_1_/*c*) (crystallographic details are presented in Table S2‐S4).

**FIGURE 2:**
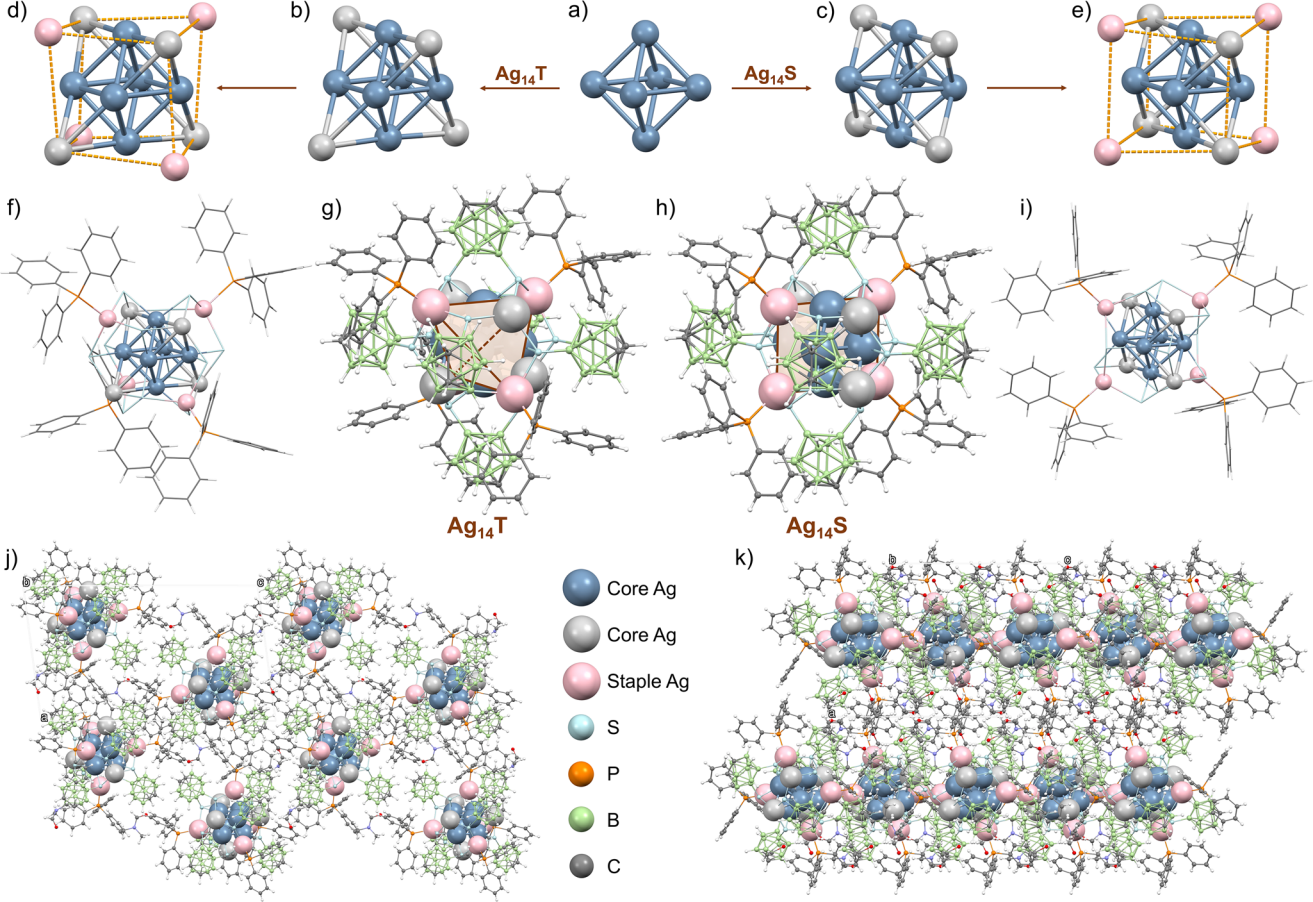
Difference in crystal structures and their bonding pattern. (a) Octahedral Ag_6_ core. (b,c) additional four other Ag atoms arranged in either tetrahedral (Ag_14_T) or square planar (Ag_14_S) fashion, respectively. (d,e) the distorted fcc‐Ag_14_ metal core–shell structures of the respective Ag_14_T and Ag_14_S NCs. (f), (i) Site‐specific positioning of TPP. (g,h) Overall structure of Ag_14_T and Ag_14_S NCs. (j,k) the differences in packing of Ag_14_T and Ag_14_S NCs, respectively.

A comprehensive crystallographic examination confirms that both Ag_14_T and Ag_14_S NCs adopt a clear core–shell architecture, with the inner metallic core distinguished from the outer shell based on direct connectivity of the latter to the surface‐protecting ligands. In both species, the octahedral Ag_6_ central core is similar (Figure 2a). Beyond that, their structural divergence becomes evident. In Ag_14_T, four silver shell atoms cap four faces of the octahedron, which have no shared edges, in a tetrahedral arrangement, resulting in a pyramidal metallic framework (Figure 2b). Conversely, in Ag_14_S, the four silver atoms cap two pairs of coplanar faces of the octahedron, which form a “belt”, in a square‐planar configuration, generating a rod‐like extended geometry (Figure 2c). In both species, the intra‐core Ag–Ag separations range from 2.70 to 2.95 Å, consistent with metallic bonding and in close agreement with the Ag–Ag distance in bulk silver (2.89 Å) (Figure S2). The outer shell is completed by an additional four silver atoms that cap the remaining four faces of the Ag_6_ octahedron, forming a distorted cubic arrangement (Figure 2d). These outer “staple” silver atoms are positioned 3.30–3.40 Å from the nearest core atoms, distances characteristic of weaker metallic connectivity (Figure S2). The distortion of this outer cube is attributable to the binding of TPP ligands, which exert an outward pull, displacing them from their idealized positions. This effect is apparent in both Ag_14_T and Ag_14_S (Figure 2f,i). The P⋯P distances between face‐diagonally opposite phosphorus atoms coordinated to silver atoms are 10.702–10.840 Å in Ag_14_T and 10.681 Å in Ag_14_S (Figure S3). The CBDT ligands are anchored to the staple silver atoms, with average staple Ag─S bond lengths of 2.67 and 2.69 Å for Ag_14_T and Ag_14_S, respectively (Figure S2). Thus, while both structures exhibit analogous core, staple, and ligand arrangements, the distinct geometries induced by differential TPP orientations result in two structurally and functionally unique NC systems (Figure 2g,h). These systems not only establish the sensitivity of Ag_14_ clusters to surface‐ligand coordination but also highlight the capability of outer phosphine ligands to direct and distort the geometry of inner metallic frameworks [56].

Analysis of unit‐cell packing (Figure 2j,k and Figure S4) reveals two molecules per unit cell for both clusters (Z = 2). Solvent‐accessible regions in the lattices contain two and five DMF molecules surrounding Ag_14_T and Ag_14_S NC, respectively. The crystal packing is stabilized by an array of weak non‐covalent interactions, most notably C─H···π, C─H··C─H and B─H···π interactions between the coordinated solvent molecules and the NC frameworks (Figure S5). Furthermore, B‐H···π contacts between CBDT and TPP ligands contribute additional stabilization within the lattice. The centroid‐to‐centroid distances between adjacent Ag_14_T and Ag_14_S NCs are 17.467 and 17.490 Å, respectively, indicating nearly identical intercluster separations in both NCs (Figure S6). Subtle differences in molecular packing can be directly attributed to the distinct orientations of TPP ligands in each NC, further underscoring the structural influence of surface‐ligand geometry. The absence of any detectable counterions indicates that both Ag_14_T and Ag_14_S are overall neutral species, with intermolecular cohesion arising predominantly from van der Waals and other weak supramolecular forces. From a valence electron‐counting perspective, the free‐electron count for each cluster is given by [(14 × 1) − (6 × 2) − (4 × 0)] = 2, corresponding to a superatom electronic configuration [57, 58]. The experimental Powder‐XRD patterns of both Ag_14_T and Ag_14_S agree well with the simulated patterns and are clearly distinct from each other, confirming the phase purity of the bulk samples without any detectable coexistence of the two isomers (Figure S7).

### 2.3 Additional Characterization

Electrospray ionization high‐resolution mass spectrometry (ESI‐HR‐MS) studies were performed to verify the molecular compositions of these clusters in DMF solution. No peaks were observed for both the clusters in the positive as well as negative ion modes, which is due to their neutral character, as suggested by SCXRD and free‐electron count. Both clusters were then ionized using Cs^+^ attachment. A single peak at *m*/*z* 2881.31 with (1+) charge state corresponding to [Ag_14_(CBDT)_6_]Cs^+^ was observed in positive ion mode for Ag_14_T and Ag_14_S clusters, as shown in Figure 3. The isotopic distribution of the experimental peak matches well with the simulated spectrum (inset of Figure 3). The complete loss of TPP in both cases is consistent with our observation of solvent‐specific tunable TPP attachment in the crystalline state. Similar types of TPP‐detached MS peaks have often been observed in the literature, confirming its labile nature [56, 59, 60]. The UV–vis absorption spectra of the solid NCs show broad absorption features (Figure S8). The onset of the absorption band edge was observed ∼560 nm for Ag_14_S, and it shifted to ∼530 nm for Ag_14_T. The UV–vis absorption spectra of Ag_14_T and Ag_14_S recorded in DMF and in DMF:MeOH (1:1, v:v) are essentially identical, with absorption maxima at 400 and 465 nm, respectively, due to identical electronic structure (Figure S9). In addition, the ^31^P NMR spectra in the respective solvents indicate that TPP remains coordinated to the silver atoms in both the NCs (Figure S10).

**FIGURE 3:**
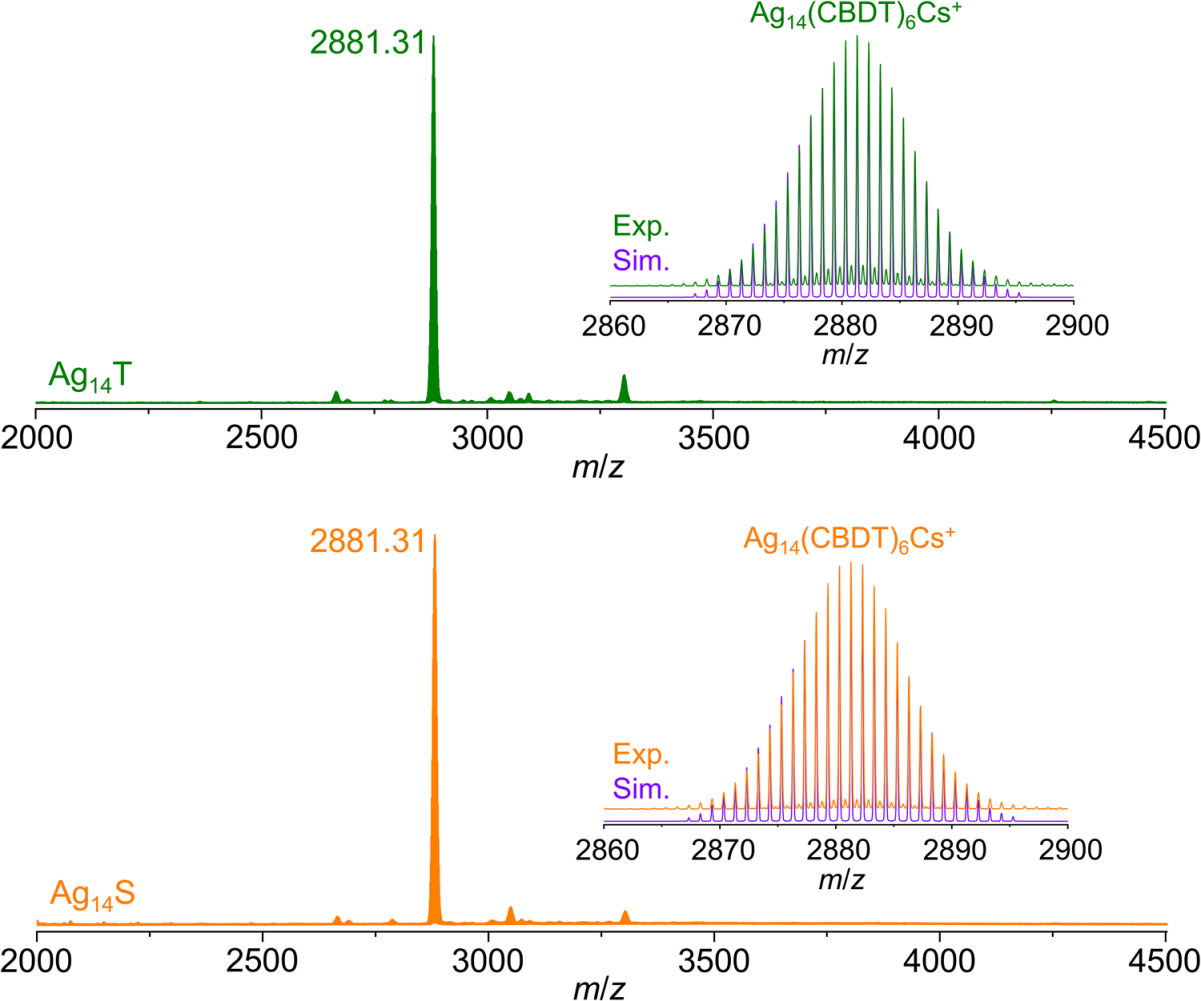
Full range ESI‐mass spectrum of the Ag_14_T (green) as well as Ag_14_S (orange) clusters in DMF solution. The inset shows the exact matching of the isotopic distributions of the experimental and simulated spectra. A complete dissociation of TPP ligands was observed in solution.

### 2.4 DFT Calculations of Optical Absorption Properties

To elucidate the relationship between structure and optical response, two geometries of Ag_14_ NCs were investigated, as their distinct geometries lead to notable differences in electronic and optical characteristics. The electronic structures were analyzed using DFT. The projected density of states (PDOS) was obtained by decomposing the Kohn–Sham states into spherical harmonic components centered at the center of mass of the cluster, revealing the positions and symmetries of the superatomic states (Figure 4c,d and Figure S11). Both geometries exhibit 2e^−^ superatomic character, with the highest occupied molecular orbital (HOMO) having S symmetry. The LUMO orbitals in both isomers are localized to TPP ligands, and the P‐symmetric superatom orbitals are found at a slightly higher energy. Decomposition of the PDOS into contributions from Ag, CBDT, and TPP shows that the HOMO is primarily localized on the Ag core and CBDT ligands, while the LUMO is distributed mainly over the TPP ligands for both clusters (Figure 4b). The isomers have slightly different HOMO‐LUMO energy gaps, decreasing from 2.71 eV (Ag_14_T) to 2.55 eV (Ag_14_S) (Figure 4c,d).

**FIGURE 4:**
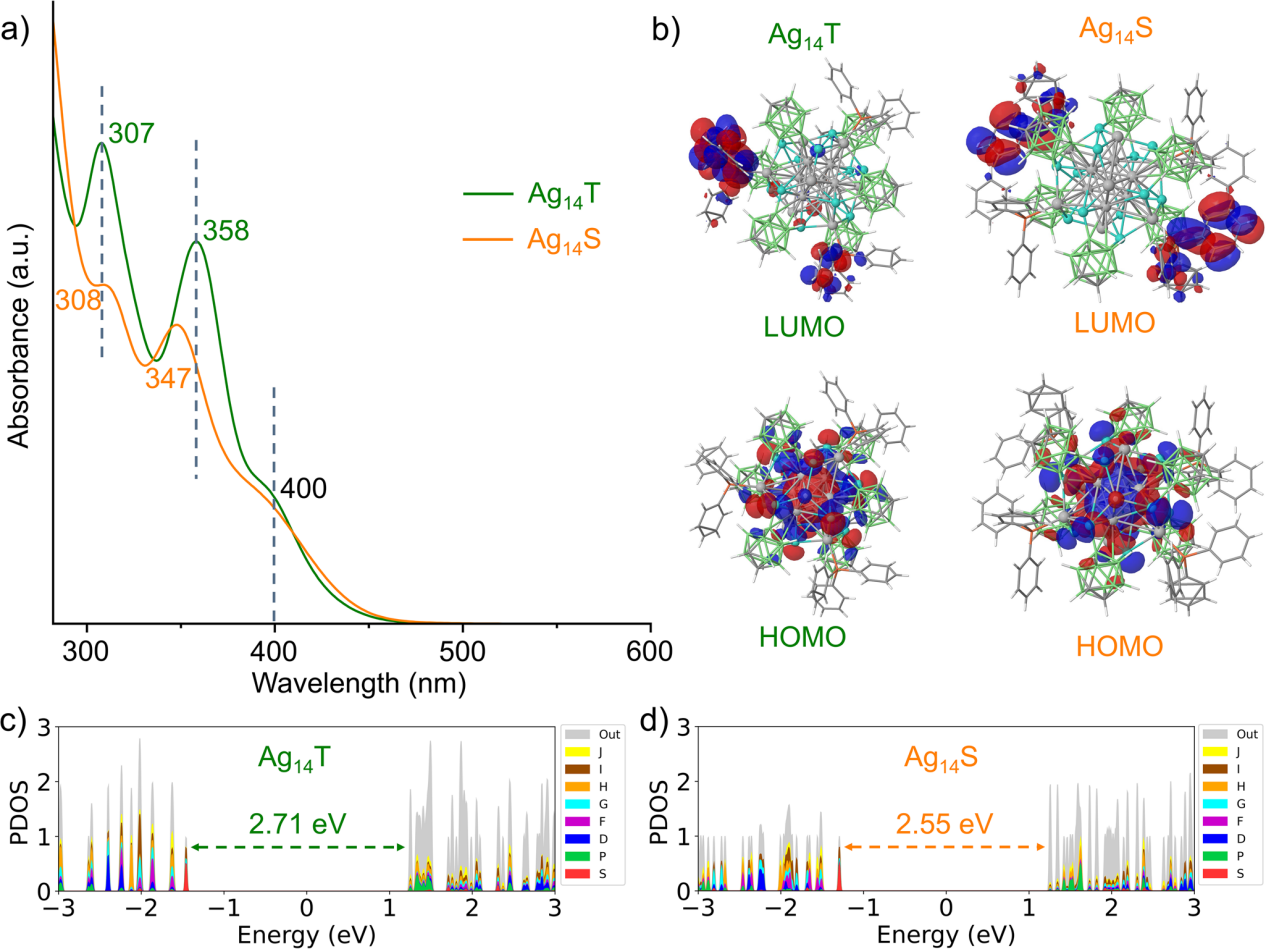
(a) TD‐DFT simulated optical absorption spectra of Ag_14_T and Ag_14_S NCs, showing the shift of high‐energy bands. (b) Visualization of HOMO and LUMO orbitals of Ag_14_T and Ag_14_S NCs. (c,d) represent the comparative HOMO‐LUMO energy gap of Ag_14_T and Ag_14_S NCs, respectively. The DOSs are centered on zero, and the magnitude of the gap is presented.

The optical absorption spectra were calculated using linear‐response time‐dependent density functional theory (LR‐TDDFT) with the PBE exchange‐correlation functional (Figure 4a). Both isomers show a weak absorption shoulder at about 400 nm, followed by clear peaks at 358 and 307 nm (Ag_14_T) as well as 347 and 308 nm (Ag_14_S). The optical gap of Ag_14_S is slightly smaller than that of Ag_14_T, following the trend in HOMO‐LUMO energy gaps.

Additional weaker absorption bands are observed near 400 nm and 307–308 nm in both the clusters. To clarify the nature of these transitions, dipole transition contribution maps (DTCMs) were computed using time‐dependent density functional perturbation theory (TD‐DFPT) (Figures S12 and S13). The first absorption feature around 400 nm originates mainly from the superatomic S orbital (HOMO in both isomers) to superatomic P orbitals (LUMO+1 in Ag_14_T, LUMO+2 in Ag_14_S). The higher‐energy bands correspond to mixed metal‐ligand excitations. The DFT and LR‐TDDFT analyses demonstrate that geometric rearrangement from Ag_14_T to Ag_14_S NC structure modifies the frontier orbital distribution and charge‐transfer interactions between the silver core and ligands.

### 2.5 Tunable Photoluminescence Properties of the Isomers

Despite their identical molecular composition, Ag_14_T and Ag_14_S NCs show surprisingly distinct PL properties in the solid crystalline state, whereas in solution, both clusters show identical emission behavior due to rapid rotational behavior (Figure S14). The solid crystalline Ag_14_T and Ag_14_S emit green and red luminescence at room temperature, respectively, due to the restriction of the rotational relaxation (as shown in the optical images in Figure 5c,d). Upon photoexcitation using ca. 320 nm, Ag_14_T shows an emission band centered at 480 nm (Figure 5a) at 295 K. It decays monoexponentially with a decay constant of τ = 4.7 µs (Figure S14) and has a quantum efficiency of 7% (determined in an integrating sphere upon excitation at λ_exc._ = 320 nm). Decreasing the temperature till 3.2 K notably narrows that band, increases its integral intensity by ∼15‐fold with a 10 nm redshift of the emission band to 490 nm (as estimated from the temperature‐dependent emission spectra, thus approaching ∼100% quantum yield), and slows down its decay to τ = 72 µs (Figure S15). The emission can be assigned to phosphorescence of the excited triplet state in Ag_14_T, which likely has a metal core to ligand(s) charge transfer (MLCT) character. In contrast to Ag_14_T, in the solid state at room temperature, Ag_14_S emits a moderately bright red emission at ca. 720 nm. Its quantum efficiency amounts to 12% (λ_exc._ = 320 nm). The excitation spectrum has an onset at ca. 560 nm and thus complies with the red‐yellow color of solid Ag_14_S. However, the PL dramatically and reversibly changes by decreasing the temperature below ∼200 K (Figure 5b): two additional emission bands develop at low temperatures around 450 and 595 nm. All three bands decay on the timescale of tens of microseconds (only moderately dependent on the temperature, Figure S16) and thus can be assigned to phosphorescence. The total emission intensity of Ag_14_S increases by ∼10‐fold below ∼100 K as compared to the PL at room temperature. Correspondingly, both Ag_14_T and Ag_14_S are very efficient emitters at low temperatures (with estimated PL quantum yield approaching unity). The emission band at 450 nm of Ag_14_S shows a similar ligand‐based PLE spectrum to that observed for Ag_14_T and thus can be related to a similar excited state configuration/ emissive transition as in Ag_14_T. However, the PL excitation spectra related to the 595 and 700 nm emission bands of Ag_14_S are distinct and involve low‐energy excitations. We have schematically illustrated in Figure 5 the excited state energy levels for Ag_14_T and Ag_14_S clusters with a (nearly) single‐band phosphorescence from the excited triplet state of Ag_14_T (T_1_ = 2.53 eV) and a multi‐band phosphorescence of Ag_14_S at 3.2 K (at 1.77, 2.08 and 2.76 eV). We tentatively assign such complicated PL behavior of Ag_14_S to several possible triplet state configurations with slightly different geometries of the silver core (and notably different triplet energies). Temperature‐dependent electronic relaxation processes as well as changes in solid‐state packing and in the geometry of the silver core, driven by subtle ligand reconfigurations during temperature cycling, may govern the relative population of specific excited states.

**FIGURE 5:**
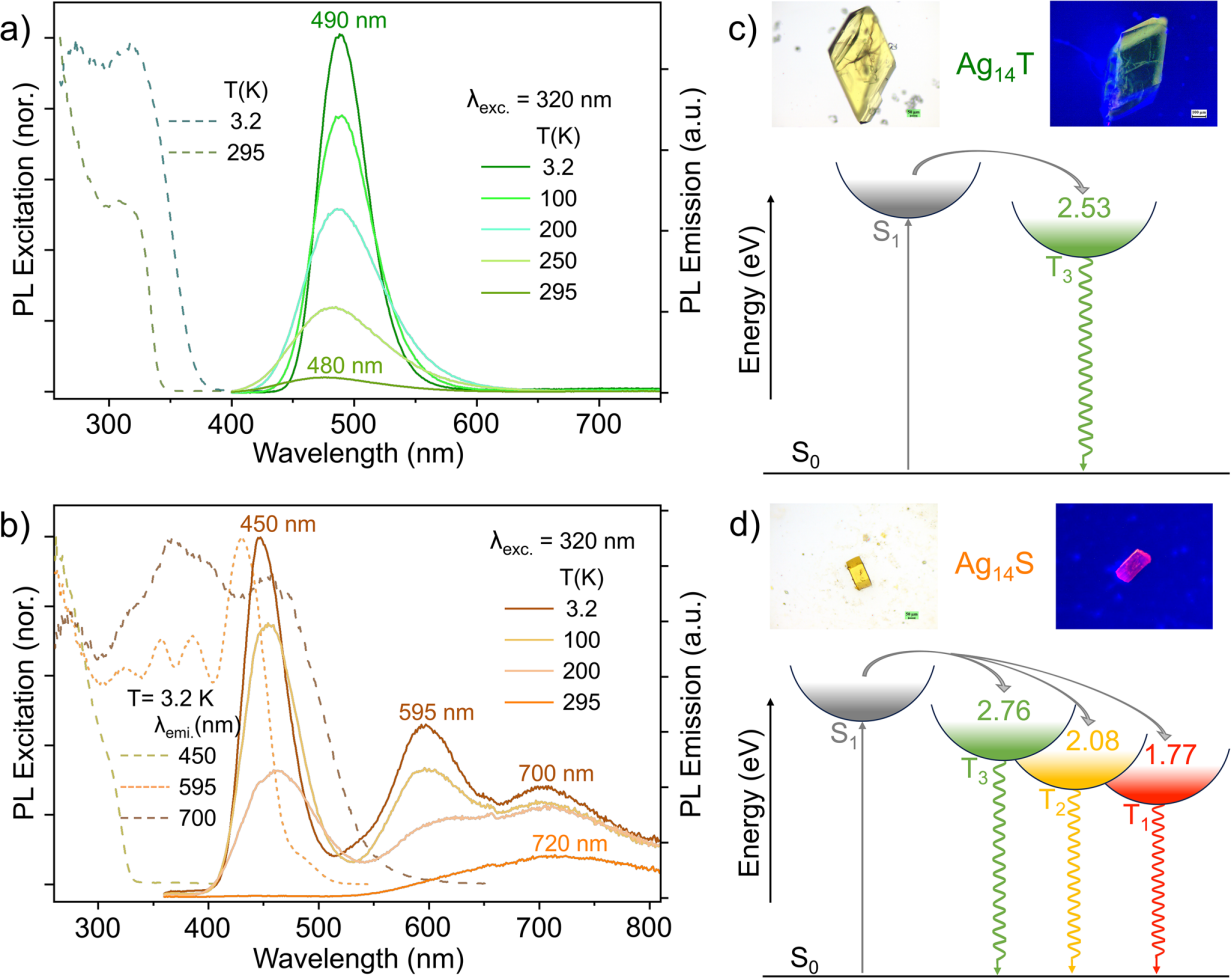
The PL excitation and emission spectra of (a) Ag_14_T and (b) Ag_14_S NCs in solid crystalline state at temperatures between 3.2 and 295 K. Schematic illustration for the triplet excited state and radiative relaxation mechanism of (c) Ag_14_T and (d) Ag_14_S NCs at 3.2 K. Inset shows the optical microscopic image of (c) Ag_14_T and (d) Ag_14_S crystals in normal light and under UV light at room temperature.

### 2.6 Solid‐State Packing Resolved Through Nanoindentation Studies

To gain further insight into the solid‐state packing, nanoindentation studies were conducted (Figure 6 and Figures S17–S22). Single crystals with clean surfaces were mounted on a glass coverslip using super glue, and it was further glued on top of a steel stub. The exposed faces of crystals Ag_14_T (101) and Ag_14_S (110) were indented, with peak loads of 1, 2, and 5 mN and with a loading/unloading rate of 0.2, 0.5, and 1 mN s^−1^, respectively, including a hold interval of 2 s before unloading (Figure 6a). Representative load‐displacement (P‐h) curves and 3D scanning probe microscopy (SPM) images of the indent impressions at 5 mN load on the crystal faces of Ag_14_T and Ag_14_S are shown in Figure 6b. Data suggest that the faces of Ag_14_S are marginally stiffer and harder than those of Ag_14_T. The smooth nature of the loading curves negates any sudden displacement bursts or pop‐in events, which are generally seen in the crystals with layer‐like structures [49]. This is evident from the crystal packing of Ag_14_T and Ag_14_S, since the indentation was done on top of the (101) and (110) faces for Ag_14_T and Ag_14_S crystals, respectively. The *E* and H values have been extracted using the standard Oliver–Pharr (O‐P) method [61]. The obtained *E* values of Ag_14_S and Ag_14_T are 8.48 (σ ≈ 0.9) and 6.05 (σ ≈ 1.23) GPa at 1 mN load, and 7.26 (σ ≈ 0.78) and 6.19 (σ ≈ 0.96) GPa at 5 mN load, respectively (Table S1). In contrast, *H* values of Ag_14_S and Ag_14_T are 534 (σ ≈ 110) and 340 (σ ≈ 79) MPa at 1 mN load, and 373 (σ ≈ 63) and 344 (σ ≈ 49) MPa at 5 mN load, respectively. The slightly stiffer and harder nature of Ag_14_S than Ag_14_T is consistent with the higher *E* and *H* values in Ag_14_S compared to Ag_14_T. The supramolecular crystal packing of Ag_14_T shows a “parallel lamellar” arrangement of clusters perpendicular to the (101) plane, separated by CBDT and TPP ligands (Figure 6c), whereas Ag_14_S exhibits a “slanted layer”‐like packing perpendicular to the (110) plane (Figure 6d). A lower indentation depth under identical applied pressure for Ag_14_S indicates greater molecular rigidity due to slanted layer packing and greater molecular rigidity than in Ag_14_T. For a quantitative analysis to establish the relation between crystal packing and the observed mechanical properties, we calculated the void ratio for both crystal systems using CIF files in the Mercury software (Figures S23 and S24). A lower value of void ratio for the Ag_14_S system (4.7%) indicates a more compact and stiffer crystal lattice compared to the Ag_14_T crystal (16.1%), which is reflected in their mechanical properties. However, both the crystals displaying mechanical properties are suitable for integration into solid‐state devices. Quantitative assessment of nanomechanical properties of numerous metal‐nanocluster crystals may enable us to make broader conclusions on the design of future nanodevices for opto‐electronic applications.

**FIGURE 6:**
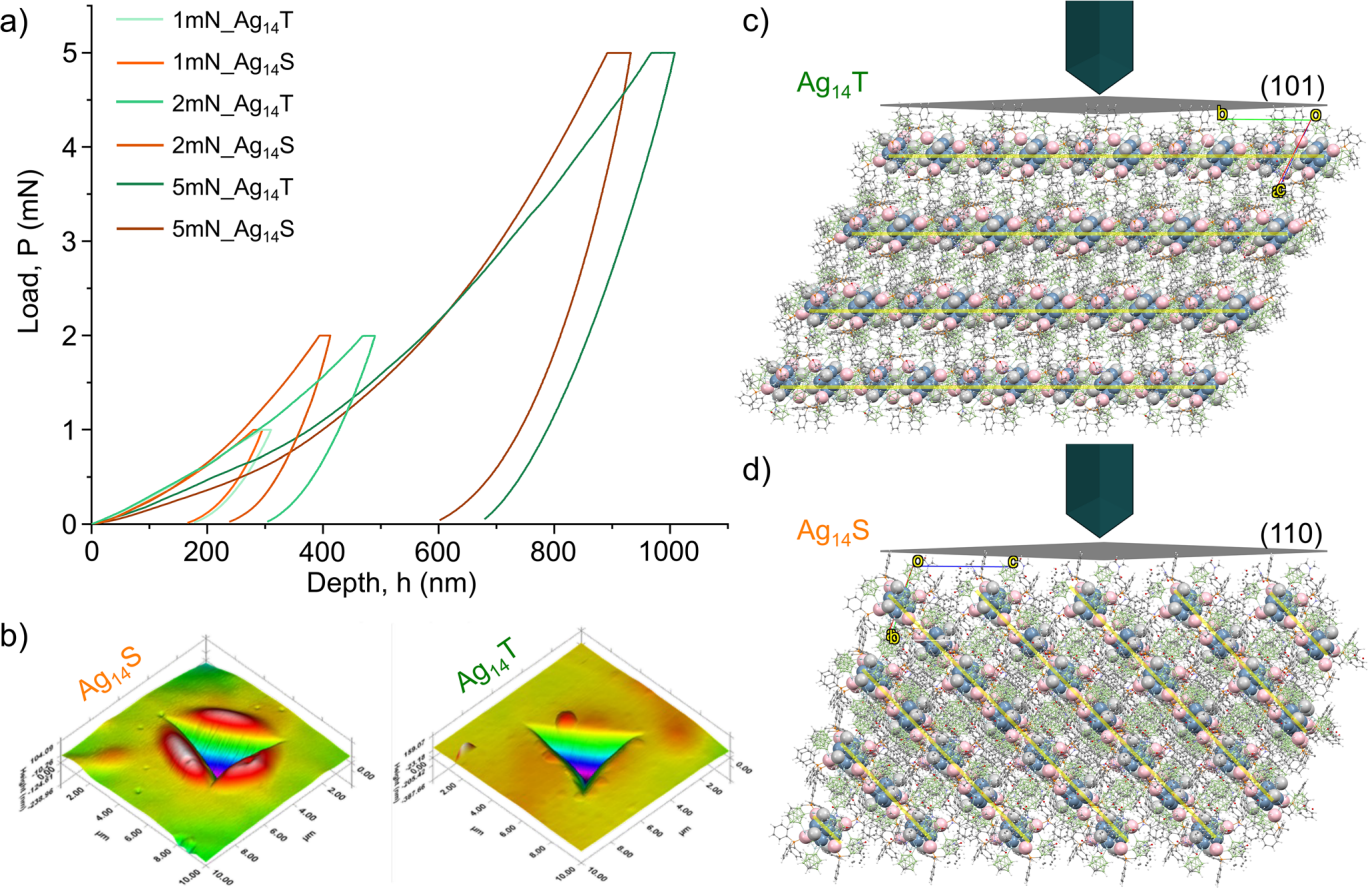
(a) Representative load (*P*)‐displacement (*h*) curves at different applied loads for both the Ag_14_ crystals. (b) 3D indent impressions after nanoindentation using 5 mN applied load on Ag_14_ crystals. Extended supramolecular packing of (c) Ag_14_T showing "parallel lamellar" arrangement and (d) Ag_14_S showing "slanted layer" packing along the marked indentation planes (yellow lines representing the packing arrangements).

## 3 Conclusion

This study elucidates the synthesis, atomic‐scale structure, optical emission, and mechanical properties of two isomeric silver NCs; Ag_14_T and Ag_14_S, each comprising fourteen Ag atoms, six *ortho*‐carborane‐9,12‐dithiolate, and four TPP ligands. Through strategic modulation of the spatial arrangement of TPP ligands on the NC surface, two distinct isomers were obtained: Ag_14_T, with tetrahedral TPP coordination, and Ag_14_S, with square‐planar TPP disposition. Single‐crystal X‐ray diffraction confirmed that while both clusters share a core–shell metallic architecture, their TPP‐ligand positions induce different distortions in the outer shell, establishing a direct link between ligand binding and core structure. Density functional theory and spectroscopic analyses revealed that such ligand‐driven geometric variations critically modulate electronic transitions and PL, resulting in prominent shifts from green emission in Ag_14_T to red emission in Ag_14_S. Nanoindentation studies demonstrated that Ag_14_S exhibits greater rigidity than Ag_14_T, with both crystals displaying nanomechanical properties suitable for integration into solid‐state devices. This work thus provides a sparse example of ligand shell isomerism in metal NC, systematically describing how subtle changes in secondary ligand positioning govern core distortions, emission behavior, and mechanical attributes. These insights expand the fundamental understanding of structure‐property relationships in atomically precise nanomaterials, paving the way for rational design principles in future applications spanning optoelectronics and solid‐state devices.

## Author Contributions

**Vivek Yadav**: conceptualization, investigation, Writing – original draft, visualization, Writing – review and editing, formal analysis, project administration, data curation. **Arijit Jana**: investigation, writing – review and editing, visualization, data curation. **Maya Khatun**: investigation, writing – review and editing, visualization, data curation. **Harshita Nagar**: investigation, visualization. **Sergei Lebedkin**: investigation, writing – review and editing, visualization, formal analysis, data curation. **Amit Mondal**: investigation, writing – review and editing, visualization, formal analysis, data curation. **Swetashree Acharya**: investigation, visualization, data curation. **Sami Malola**: investigation, validation, data curation. **Sudhadevi Antharjanam**: investigation, visualization, validation, formal analysis, data curation. **Moses Egor**: investigation, visualization. **Pijush Ghosh**: investigation, writing – review and editing, visualization, validation, supervision, funding acquisition, resources. **Tomas Base**: investigation, validation, visualization, writing – review and editing, supervision, funding acquisition, resources. **Manfred Kappes**: investigation, funding acquisition, writing – review and editing, visualization, supervision, resources. **Hannu Häkkinen**: supervision, funding acquisition, investigation, writing – review and editing, visualization, validation, resources. **Thalappil Pradeep**: conceptualization, investigation, funding acquisition, writing – review and editing, visualization, validation, supervision, project administration, resources.

## Conflicts of Interest

The authors declare no conflicts of interest.

## Supporting information




**Supporting File**: advs76657‐sup‐0001‐SuppMat.pdf.

## Data Availability

The data that support the findings of this study are available from the corresponding author upon reasonable request.
