# Supplementary material for: Isomeric Ag14 Nanoclusters With Distinct Photophysical and Nanomechanical Properties
Source: Adv Sci (Weinh). 2026 Jul 20;13(57):e76657. doi: 10.1002/advs.76657 (PMC13383689; doi:10.1002/advs.76657)
Supplement: Supplementary file 1 [file ADVS-13-e76657-s001.pdf]

Supplementary Information

Isomeric Ag_{14} Nanoclusters with Distinct Photophysical and Nanomechanical Properties

Vivek Yadav[1], Arijit Jana[1], Maya Khatun[2], Harshita Nagar[1], Sergei Lebedkin[3,4], Amit Mondal[5], Swetashree Acharya[1], Sami Malola[2], Sudhadevi Antharjanam[6], Moses Egor[7,8], Pijush Ghosh[* 5], Tomas Base[* 7], Manfred Kappes[*3,4], Hannu Häkkinen[*2], and Thalappil Pradeep[*1]

1 DST Unit of Nanoscience (DST UNS) and Thematic Unit of Excellence (TUE), Department of Chemistry, Indian Institute of Technology, Madras, Chennai – 600036, India

2 Department of Physics and Chemistry, Nanoscience Center, University of Jyväskylä, FI 40014 Jyväskylä, Finland

3 Institute of Physical Chemistry, Karlsruhe Institute of Technology (KIT), Kaiserstraße 12, 76131, Germany

4 Institute of Nanotechnology, Karlsruhe Institute of Technology (KIT), Eggenstein Leopoldshafen, 76344 Germany

5 Department of Applied Mechanics and Biomedical Engineering, Indian Institute of Technology Madras, Chennai 600036, India

6 Sophisticated Analytical Instruments Facility (SAIF), Indian Institute of Technology, Madras, Chennai – 600036, India

7 Department of Syntheses, Institute of Inorganic Chemistry, The Czech Academy of Science, 1001 Husinec-Rez, 25068, Czech Republic

8 Department of Chemistry, Busitema University, Tororo, Uganda

*Corresponding author's email: pradeep@iitm.ac.in, hannu.j.hakkinen@jyu.fi, manfred.kappes@kit.edu, pijush@iitm.ac.in, tbase@iic.cas.cz

Table of Contents

Experimental details and instrumentation S2-S6

Supplementary Figures S6-S25

Supplementary Tables S26-S36

References S37

Experimental details and instrumentation

a) Synthesis of Ag_{14} NCs

Ag_{14} NCs were synthesized via direct co-reduction. $AgNO_3$ (20 mg, 0.118 mmol) was dissolved in MeOH (5 mL) and DCM (7 mL) with stirring at room temperature. After 5 min, CBDT (20 mg, 0.080 mmol) in DCM (1 mL) was added. TPP (200 mg, 0.384 mmol) in DCM (1 mL) was then introduced after another 5 min, resulting in a turbid solution that was stirred for 15 min. $NaBH_4$ (11 mg, 0.291 mmol) in ice-cold water (0.5 mL) was added dropwise, gradually turning the solution to dark brown and yielding a greenish-gray precipitate after 3 h. All steps were performed in the dark to prevent Ag oxidation. The reaction mixture was centrifuged, and the supernatant was discarded. The precipitate was washed repeatedly with MeOH to remove unreacted reagents and extracted into DMF. Crystallization from pure DMF afforded greenish yellow crystals of $Ag_{14}T$, while the DMF-MeOH mixture (1:1 v/v) yielded orangish yellow crystals of $Ag_{14}S$ after 2 weeks. Both products were isolated in high purity.

b) UV-vis absorption spectroscopy

UV-vis absorption spectra were measured in diffuse-reflectance mode using a Varian Cary 5000 UV-vis-NIR spectrometer from Agilent Technology. Microcrystalline samples are placed between two quartz plates (area 1 cm^2) after being dispersed in NVH immersion oil. The spectral data were collected using an integrating sphere at a scan rate of 600 nm/min with the Cary WinUV Scan application software.

c) Optical microscopy

Optical microscopic images (in normal light) in transmission mode were collected using a LEICA optical microscope equipped with LAS V4.8 software. Polarization of the crystals were checked using an optical polarizer. The apparent non-uniformity observed in the luminescence image of the $Ag_{14}T$ crystal in Fig. 5c is an artifact during imaging rather than an intrinsic property of the crystal. In our self-built setup to take images of luminescent crystals, the UV excitation source was directed from one side of the large crystal, which led to partial shadowing of the opposite side during image acquisition. As a result, one portion of the crystal appears more brightly emissive, while the other appears comparatively dim or non-emissive in the photograph due to shadow. This should not be interpreted as spatial heterogeneity in the photoluminescence of the crystal.

d) Mass spectrometry

High resolution mass spectra were measured using a Waters Synapt G2-Si HDMS instrument. The instrument is equipped with an electrospray ionization source, quadrupole ion trap, ion mobility separation cell, and time of flight mass analyzer. An optimized operating condition such as flow rate 10 μL/min, capillary voltage 2-3 kV, sampling cone voltage 0 V, source offset voltage 0 V, source temperature 90-100 °C, desolvation temperature 140-150 °C, and desolvation gas flow rate 400 L/h were used for all the measurements.

e) Single crystal X-ray diffraction data collection and refinement details

To elucidate the molecular crystal structure of both the NCs, Single Crystal X-ray diffraction (SC-XRD) data collection of suitable crystals was carried out on a Bruker D8 VENTURE single crystal X-ray diffractometer equipped with Mo Kα (λ = 0.71073 Å) radiation source and PHOTON II detector. The data collection was performed at an ambient temperature of ~298 K. A routine cell parameter measurement was performed initially to identify the unit cell for data collection. An optimized strategy was adopted for the full data collection by keeping an average 4-fold redundancy for the reflections. The program APEX4-SAINT[1] was used for integrating the frames, followed by a multi-scan absorption correction using the program SADABS[2]. The crystal structure was solved by intrinsic phasing method using SHELXT-2018[3] and refined by full-matrix least squares techniques using SHELXL-2019[4] software package incorporated in WingX suite[5]. Hydrogens on all carbon and boron atoms were fixed at calculated positions and refined as a riding model with C-H or B-H = 0.93 Å or 1.12 Å, Uiso(H) = 1.2 or 1.5 Ueq (C or B).

The asymmetric unit of $Ag_{14}T$, which crystallized in triclinic *P*-1 system, consists of one full molecule of the NC and two molecules of dimethyl formamide (DMF). Approximately, 17 % of unit cell volume is occupied by diffused solvent molecules. Since this diffused electron density could not be modelled properly, a SQUEEZE procedure in PLATON[6] was done to account for the electron density contribution from disordered solvent molecules. A total of 315 electrons were found in the solvent accessible volume of 1434 $Å^3$ which is equivalent of 8 DMF molecule per unit cell (4 DMF molecules in the asymmetric unit). An IUCR checkcif report was generated and explanations for B level alerts were given at the end of the cif file as Validation reply form.

The asymmetric unit the crystal lattice of $Ag_{14}S$, which crystallized in monoclinic crystal system with P 21/c space group, consists of half of the cluster molecule, 2.5 molecules of DMF and one molecule of methanol. In the crystal structure of $Ag_{14}S$, one of the phenyl rings of triphenylphosphine ligands (C7- C12 and C31-C36) was disordered with an occupancy ratio of 63:37 and 71:29 respectively. DFIX restraint was applied to C-C bond distances of these phenyl rings to achieve a target bond distance of 1.40 Å. Anisotropic displacement parameters of disordered atoms were restrained using SIMU instruction to have similar Uij components for the atoms in the disordered group. Methanol molecule in the crystal lattice was found to be disordered in the ratio of 61:39. Hydrogens on methanol solvate could not be located and refined. An IUCR checkcif report was generated and explanations for B level alerts were given at the end of the cif file as Validation reply form. Crystallographic information of the clusters are summarized in the TableS2.

f) DFT calculations

To execute the DFT computations, the GPAW software program was utilized.[7,8] With a real-space grid spacing of 0.30 Å, the converged Kohn–Sham electronic ground state was obtained using the GLLB-SC functional.[9] The PBE functional[10] was used as the exchange–correlation kernel in the linear-response time-dependent density functional theory (LR-TDDFT) as implemented in GPAW to calculate optical absorption spectra based on the ground-state wave functions. The projected density of states (PDOS) of the Kohn–Sham states were obtained by projecting them onto spherical harmonics within specific atomic spheres in order to investigate the ground-state electronic structure. After that, the sum of the selected atomic contributions and all angular momenta was used to determine the overall localization weights. Furthermore, the density of states was projected onto spherical harmonic functions centered at the center of mass in order to investigate the electronic structure. Dipole transition contribution maps (DTCMs), which are obtained from time-dependent density functional perturbation theory, were used to explain the peaks in the optical absorption spectra.

g) Photoluminescence spectroscopy

Photoluminescence (PL) measurements were performed on a Horiba Jobin Yvon Fluorolog-322 spectrometer. Temperature-dependent spectra of solid (polycrystalline) samples were recorded using a closed-cycle optical cryostat operating between 3.5 and 300 K. Solid (polycrystalline)

samples were measured as thin layer dispersions in perfluoroether oil placed between two quartz plates. All emission spectra were corrected for the wavelength-dependent response of the spectrometer and detector (in relative photon flux units). The PL measurements at decreasing/ increasing temperature were conducted for each sample at the same optical configuration and spectrometer parameters. Accordingly, the PL intensities at different temperatures can be directly compared. Emission decay traces were recorded by connecting the detector (photomultiplier) to a fast oscilloscope (LeCroy HD4104) via a load of 50, 500 or 2500 Ohm (depending on the decay time-scale) and applying a ns-pulsed nitrogen laser for excitation at 337 nm. PL quantum efficiencies of solid compounds (using the same sample preparation as above) were determined at ambient temperature with an integrating sphere out of optical PTFE, which was installed into the sample chamber of the spectrometer. The uncertainty of these measurements was estimated to be ±10%.

h) Nanoindentation experiments

The nanomechanical investigation of the metal nanocluster crystals was conducted utilizing a Hysitron TI Premier Triboindenter system, equipped with a three-sided pyramidal Berkovich diamond indenter possessing a tip radius of 150 nm. Prior to experimental measurements, instrument calibration was performed through reference indents on a standardized fused quartz substrate, ensuring measurement traceability and system compliance correction. The polymorphic $Ag_{14}T$ and $Ag_{14}S$ single crystals were glued by Fevi-kwik on glass slides and directly subjected to nanoindentation without additional processing. Due to the extremely small crystal size, nanoindentation measurements could be performed only on the dominant crystallographic faces [(101) for $Ag_{14}T$ and (110) for $Ag_{14}S$] as other orientations were not experimentally accessible despite repeated attempts. The indentation experiments were carried out by load-controlled method implementing a trapezoidal waveform profile with peak loads of 1, 2 and 5 mN, selected for their capacity to probe both near-surface and bulk mechanical response while maintaining quasi-static loading conditions. The trapezoidal function's linear loading segment (typically 5 second duration) enables controlled strain rate application, minimizing dynamic artefacts during plastic yield initiation. The unloading phase in the trapezoidal function is used to extract the elastic properties of the material by using gradual unloading of the nanoindenter from the sample. This gives an accurate load–displacement curve, which is important for analysing the material's elastic modulus.

Statistical analysis with all the data points also ensures that the data obtained are reproducible across multiple tests, making comparisons more reliable.

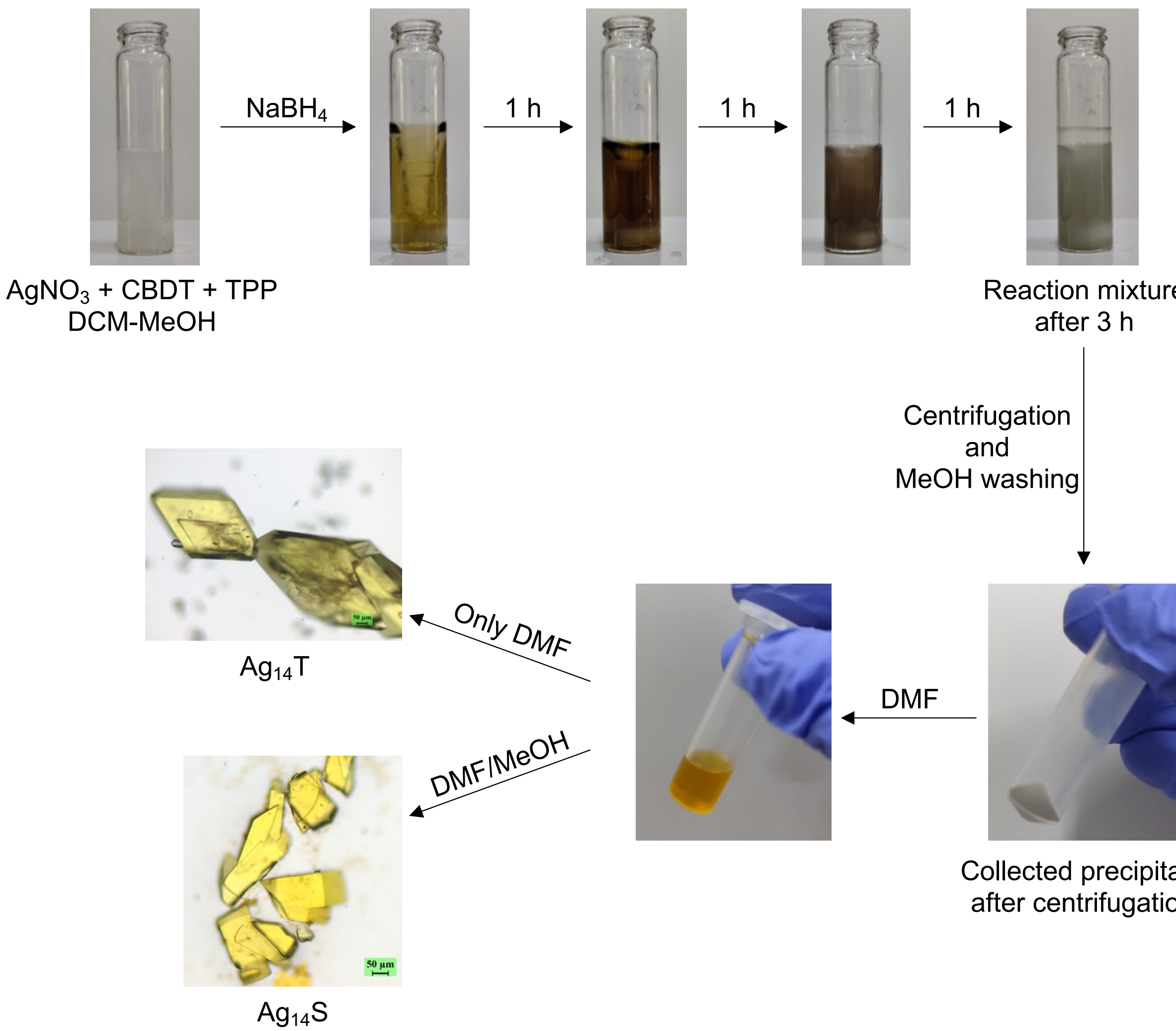

Figure S1. Schematic illustration of the time-dependent progress of the reaction, purification, and crystallization process for the Ag_{14} nanoclusters. MeOH washing purifies the crude and yielding the crystals suitable for single-crystal X-ray diffraction analysis for $Ag_{14}T$ and $Ag_{14}S$ in DMF and DMF/MeOH, respectively.

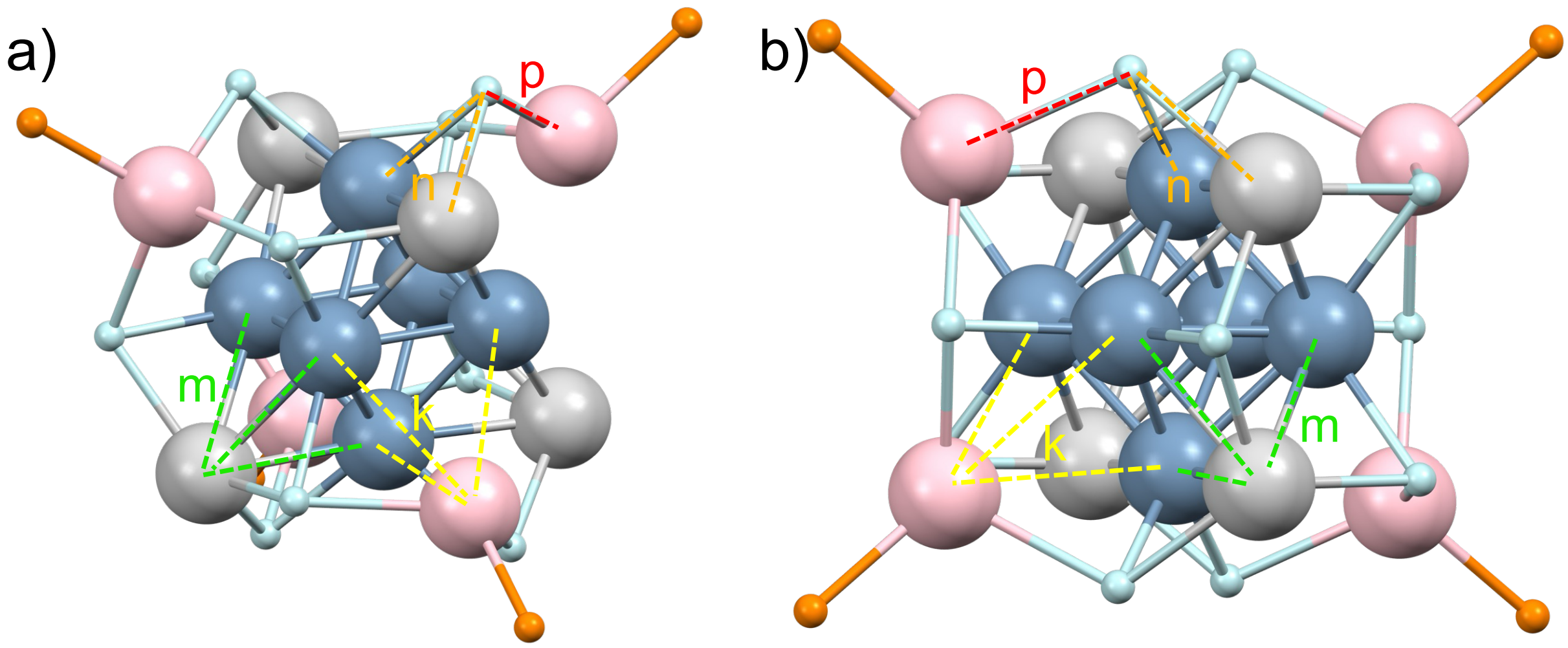

Nanocluster	Average distance (Å) between octahedron core Ag atoms and Ag of the cube attached to the octahedron core (m)	Average distance (Å) between octahedron core Ag atoms and Ag of the cube not attached (staple Ag) to the octahedron core (k)	Average distance (Å) between S atom and Ag of the cube not attached (staple Ag) to the octahedron core (p)	Average distance (Å) between S atom and all the core Ag (n)
$Ag_{14}T$	2.937	3.338	2.670	2.556
$Ag_{14}S$	2.943	3.354	2.689	2.557

Figure S2. Metal sulphur core of **a** $Ag_{14}T$ and **b** $Ag_{14}S$ shows the site-specific distortions in the core-shell structure of the desired NCs. Table shows all the average bond distances associated with the NCs.

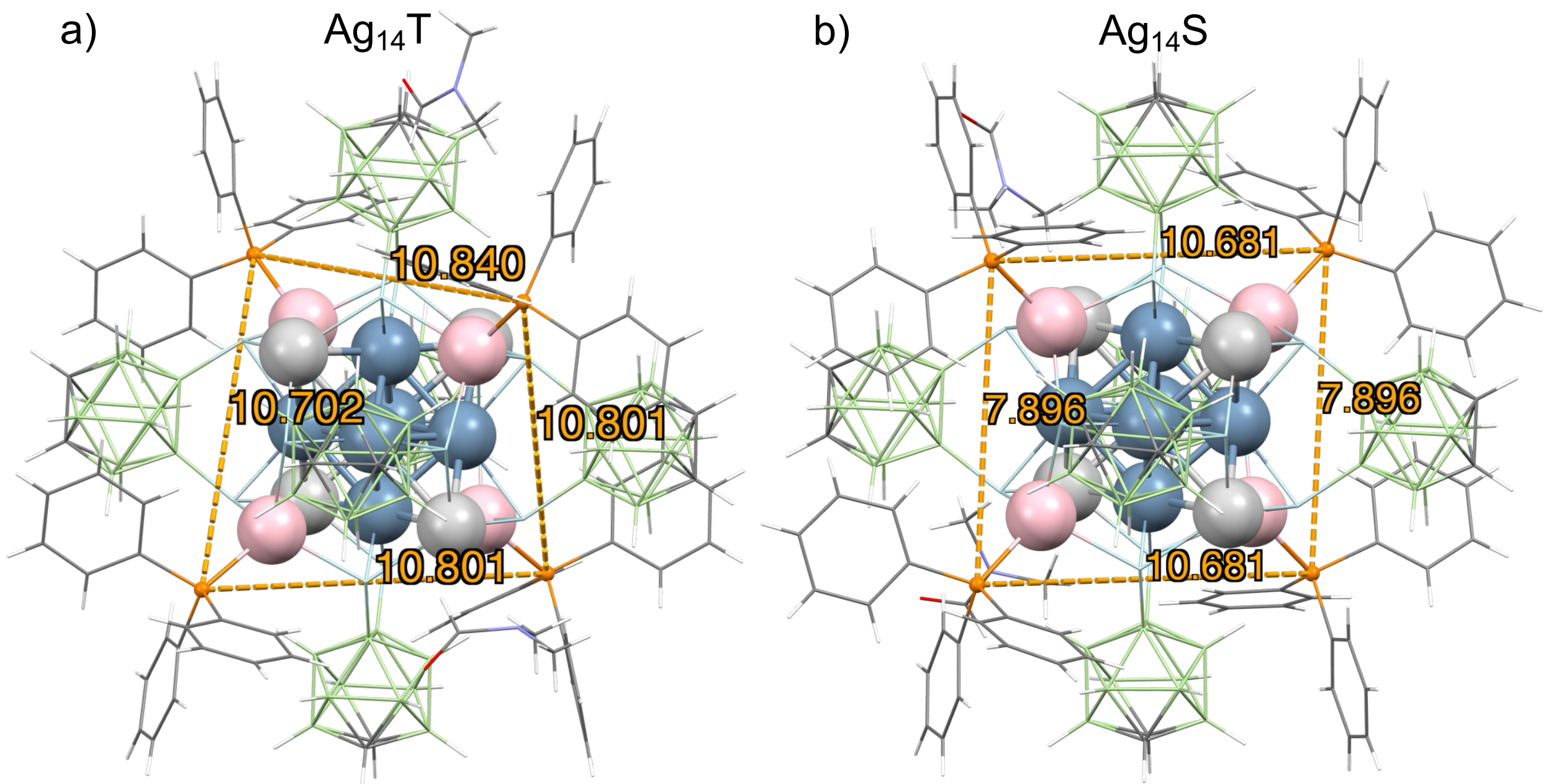

Figure S3. Comparative structural views of $Ag_{14}T$ and $Ag_{14}S$ highlighting the P···P distances between face-diagonally opposite phosphorus atoms coordinated to the silver core. The corresponding distances are a) 10.702–10.840 Å for $Ag_{14}T$ and b) 10.681 Å for $Ag_{14}S$, indicating a more contracted framework in $Ag_{14}S$. Whereas the edge phosphorous in $Ag_{14}S$ are 7.896 Å apart.

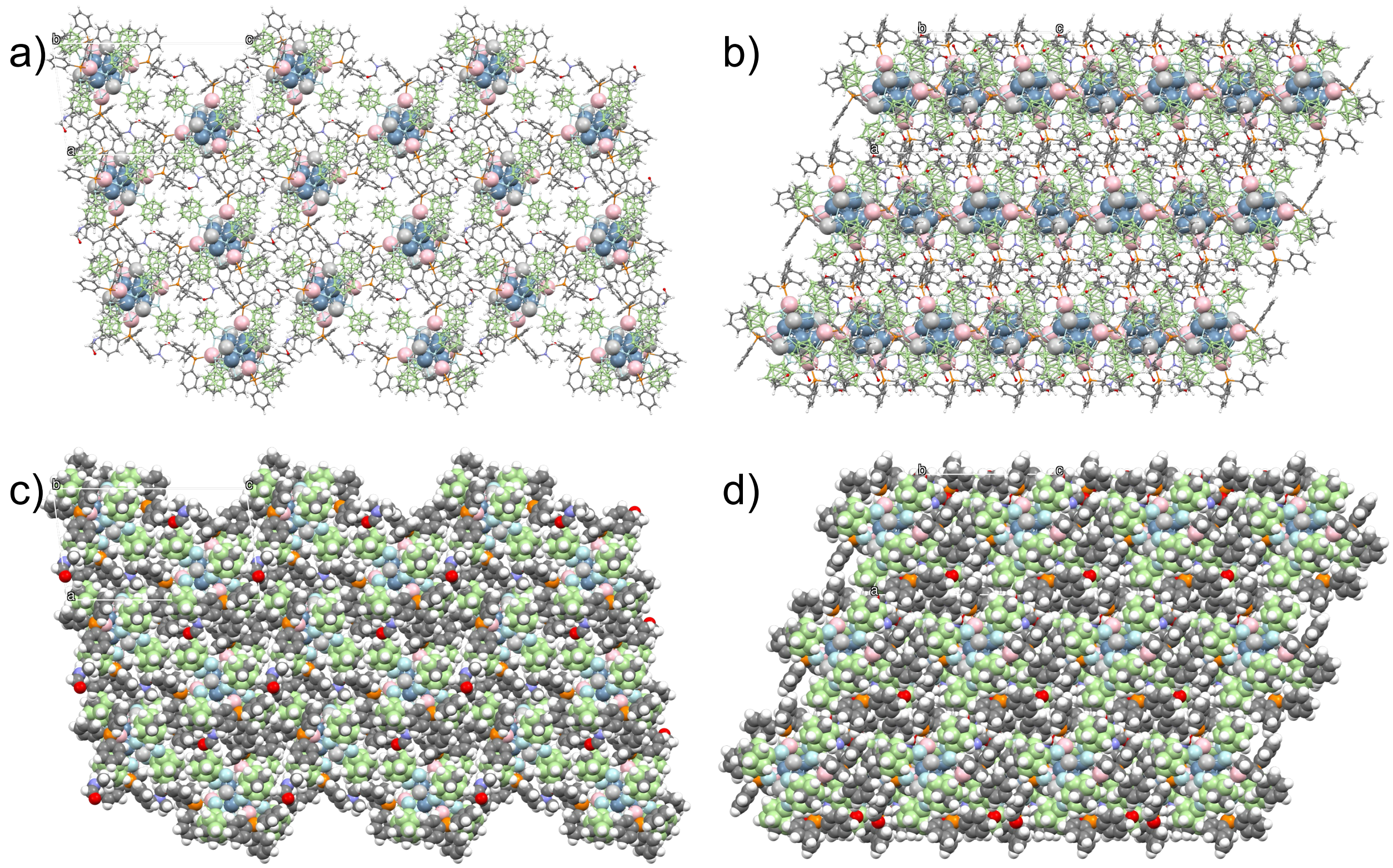

Figure S4. Extended molecular packing structures of **a, c** $Ag_{14}T$ and **b, d** $Ag_{14}S$ NCs. **a, b** show ball-and-stick representations, highlighting atomic connectivity and intercluster interactions. **c, d** depict space-filling models, emphasizing van der Waals contacts and packing density in the crystal lattice.

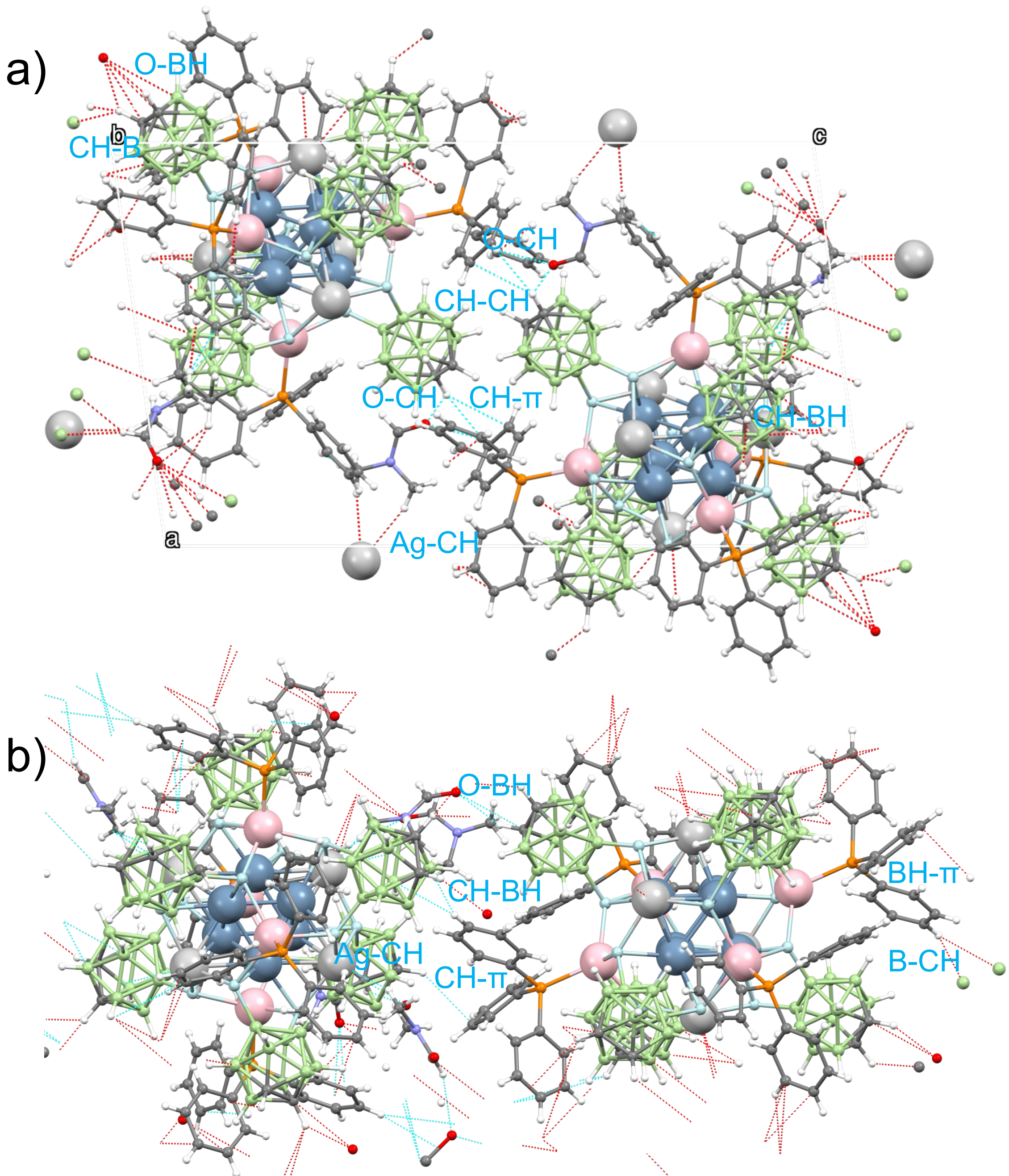

Figure S5. Intercluster interactions such as CH….π, BH….π, B….CH, BH….CH, CH….CH, B….π etc. of **a** Ag_{14}T and **b** Ag_{14}S NCs.

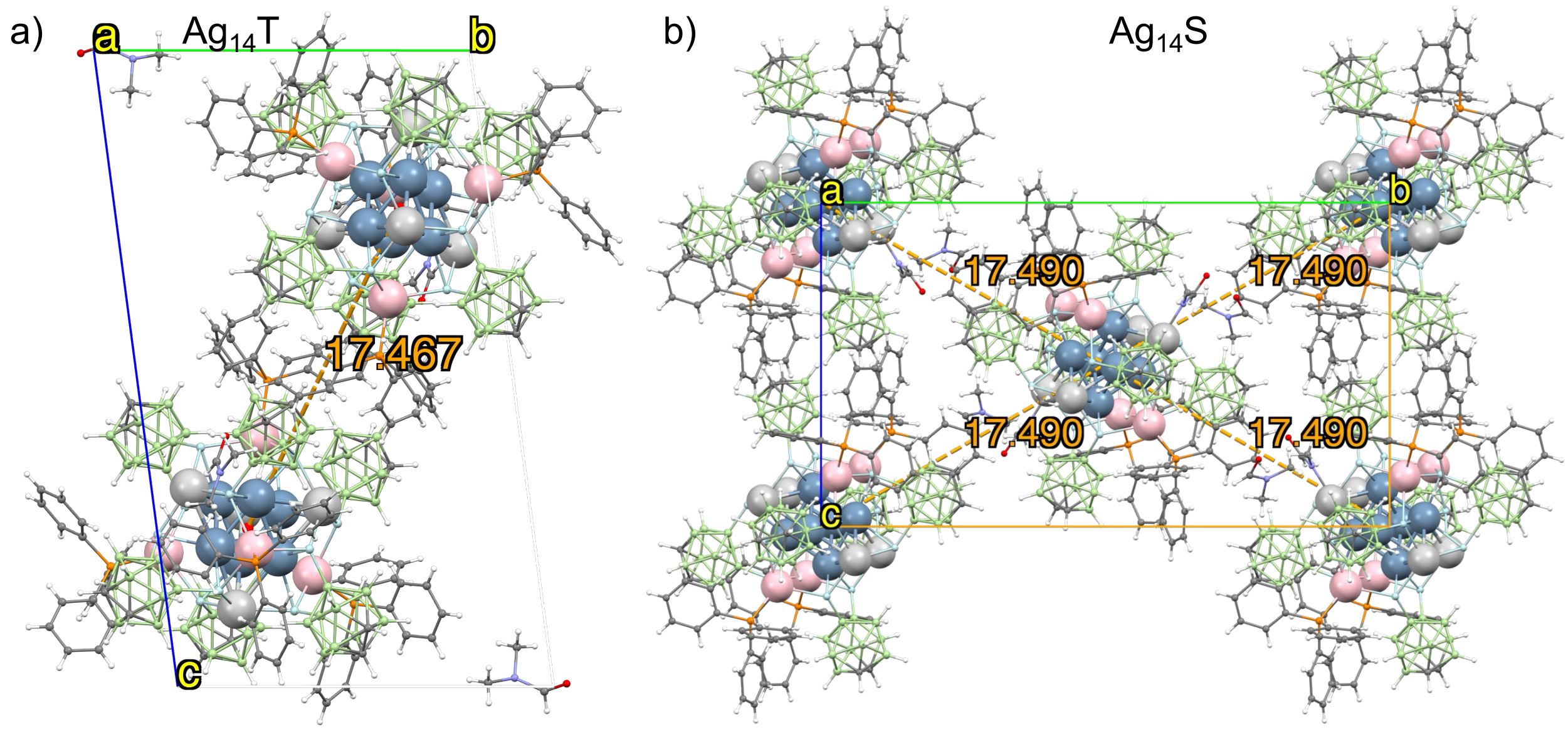

Figure S6. Centroid-to-centroid distances between adjacent a) $Ag_{14}T$ and b) $Ag_{14}S$ NCs are 17.467 and 17.490 Å, respectively, indicating nearly identical intercluster separations in both cases.

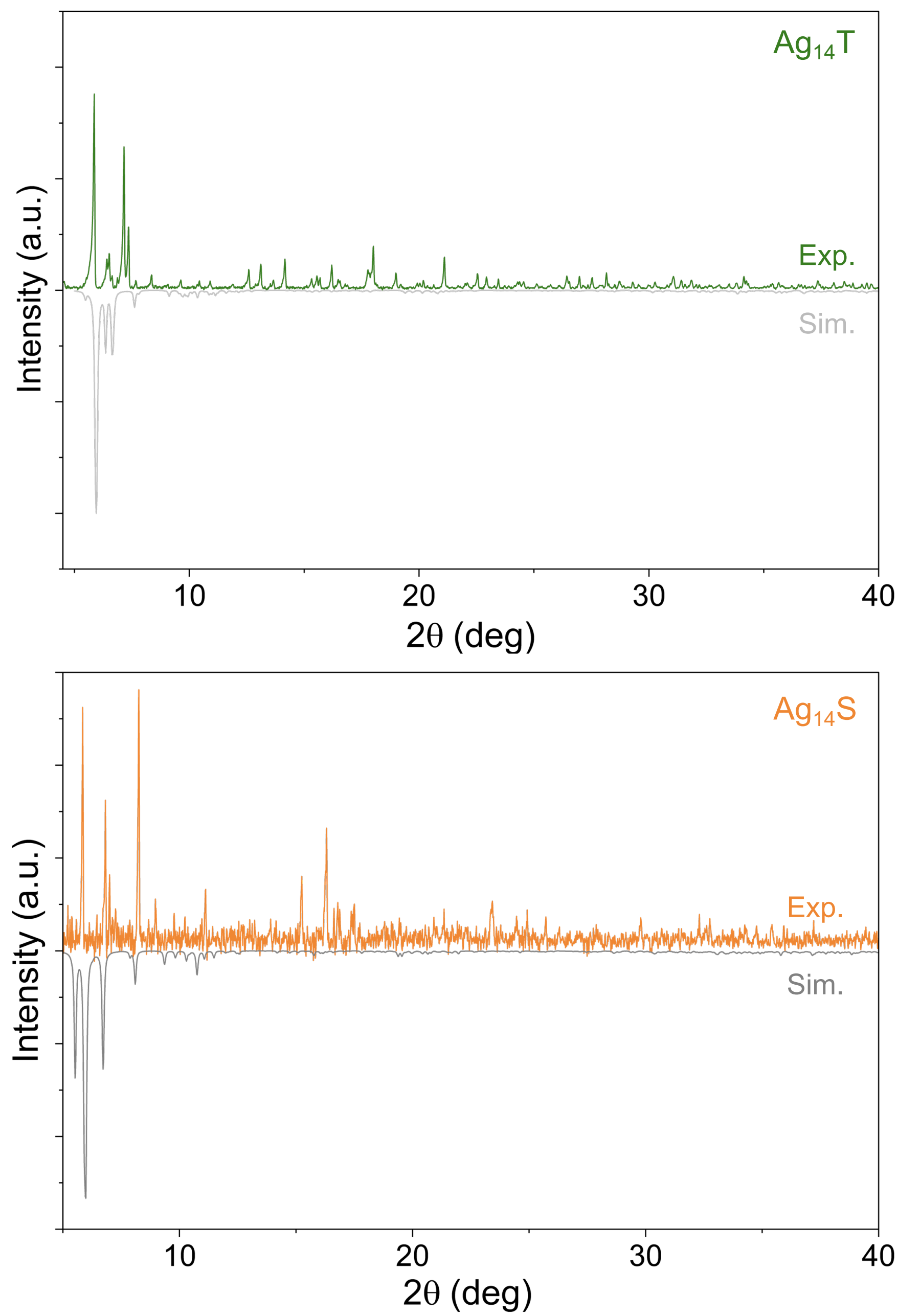

Figure S7. Experimental (Exp.) and simulated (Sim.) PXRD patterns of $Ag_{14}T$ (top) and $Ag_{14}S$ (bottom). Minor deviation of the reflexes is due to the effect of temperature and specific orientation of microcrystalline samples.

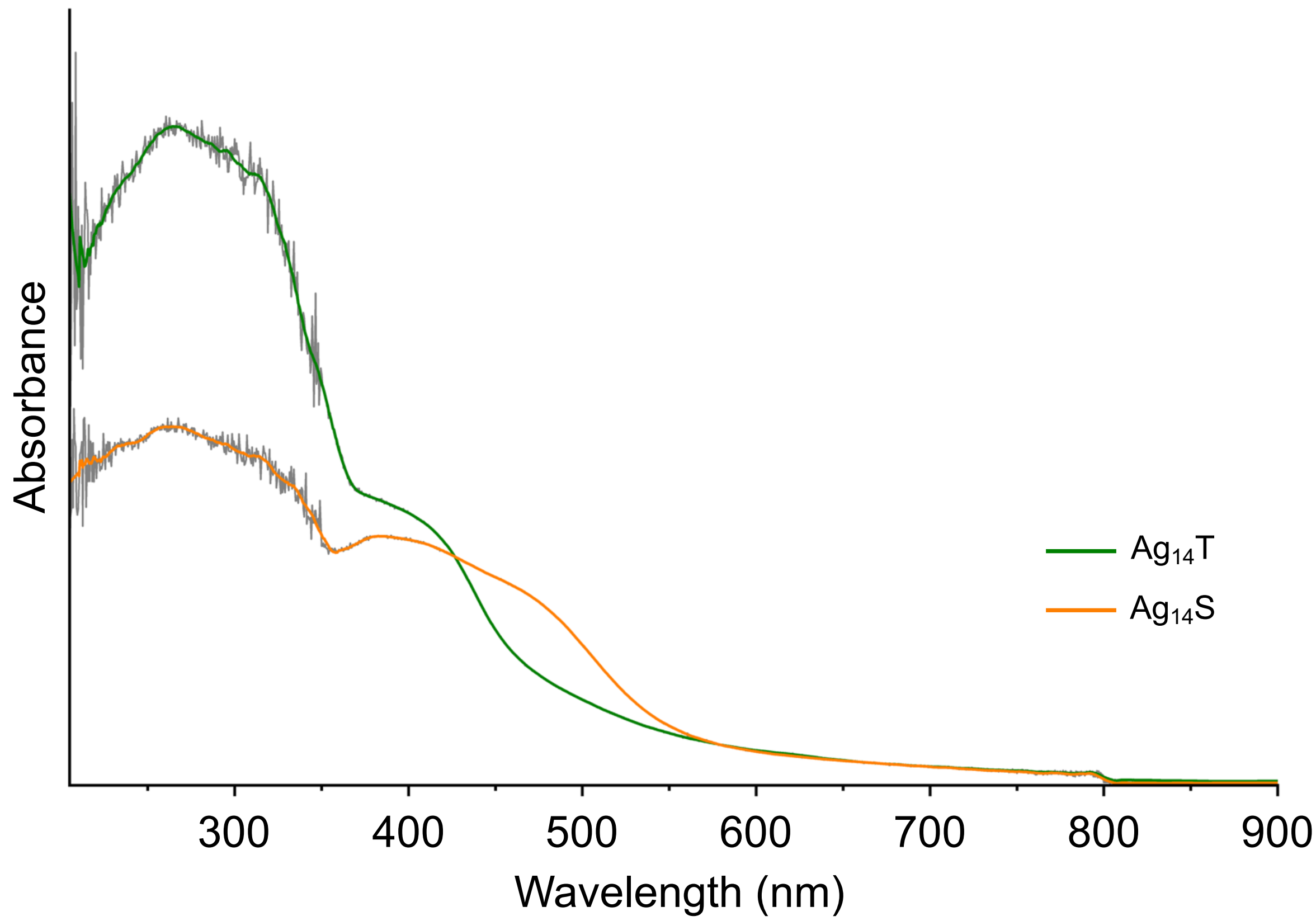

Figure S8. UV-Vis absorption spectra of $Ag_{14}T$ and $Ag_{14}S$ NCs, measured in diffused reflectance mode using microcrystalline sample of the respective clusters. The gray curves represent the raw spectra obtained directly from the instrument, while the green ($Ag_{14}T$) and orange ($Ag_{14}S$) curves correspond to the smoothed spectra derived from the raw data for improved visualization and analysis.

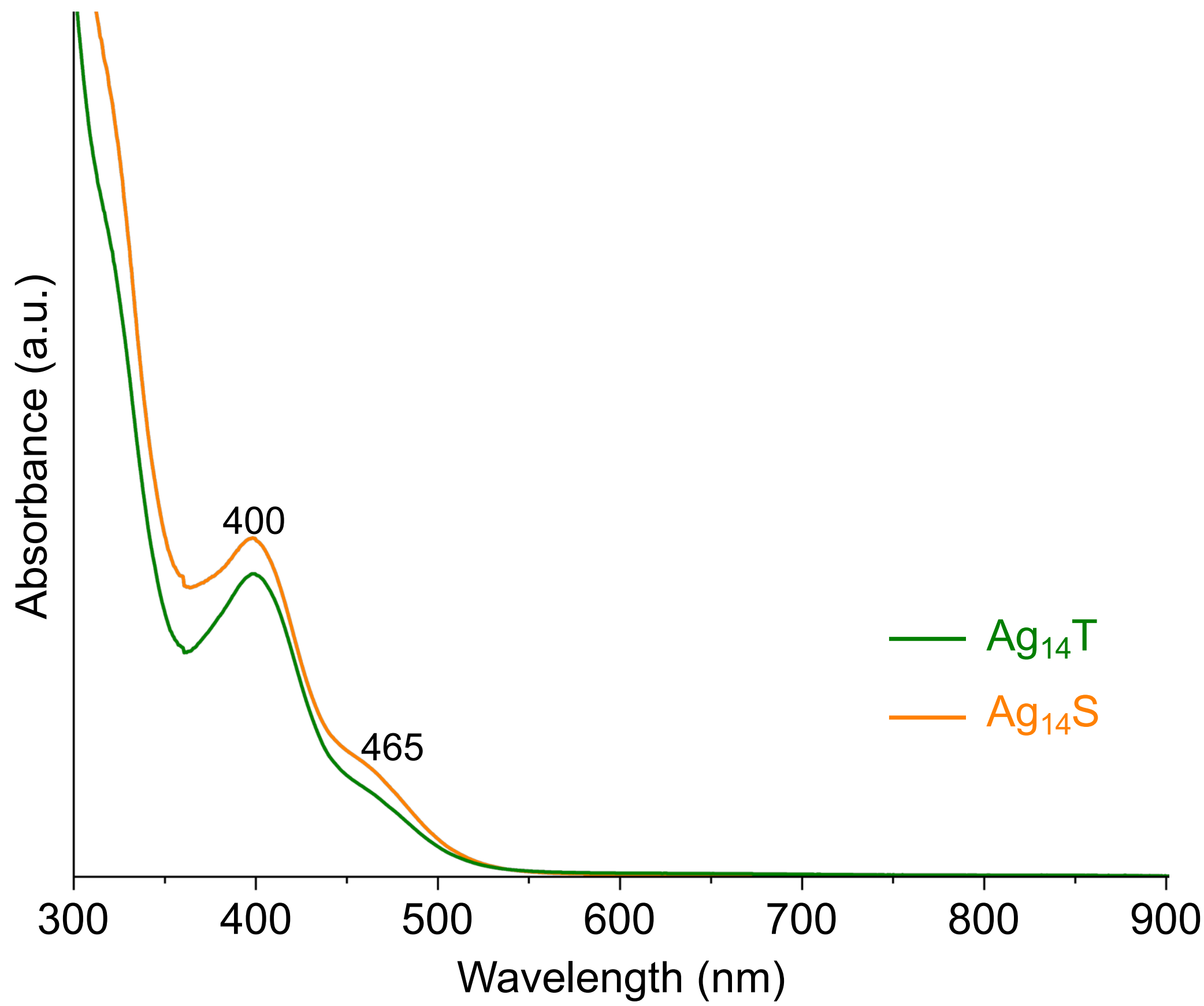

Figure S9. UV-vis spectra of $Ag_{14}T$ and $Ag_{14}S$ in DMF and DMF:MeOH (1:1, v:v) solution, respectively showing identical absorption features, with maxima at 400 and 465 nm.

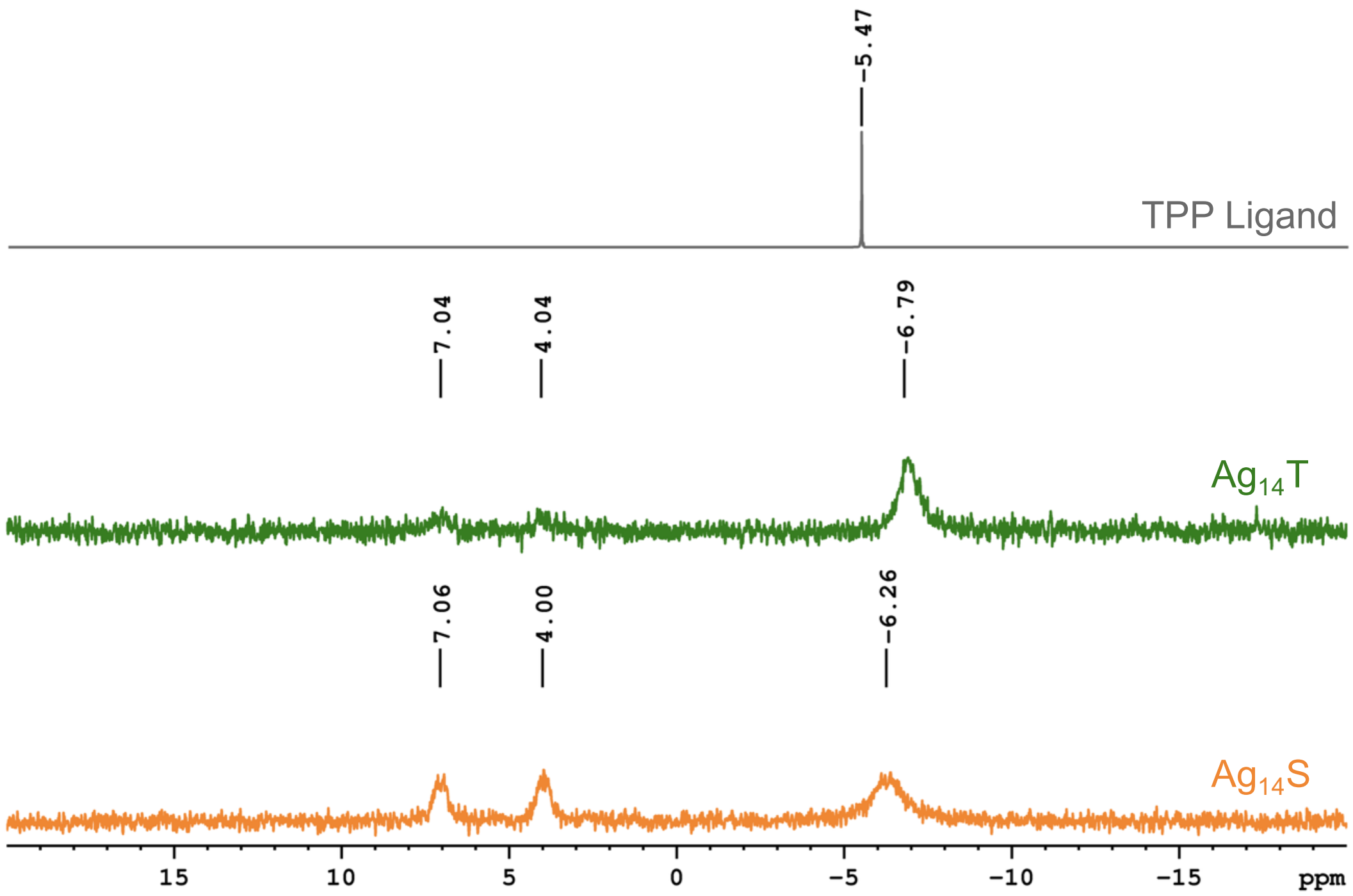

Figure S10. Comparative ^{31}P NMR showing the sharp feature of TPP ligand and the broad peaks for $Ag_{14}T$ and $Ag_{14}S$ corresponding to TPP coordinated with silver.

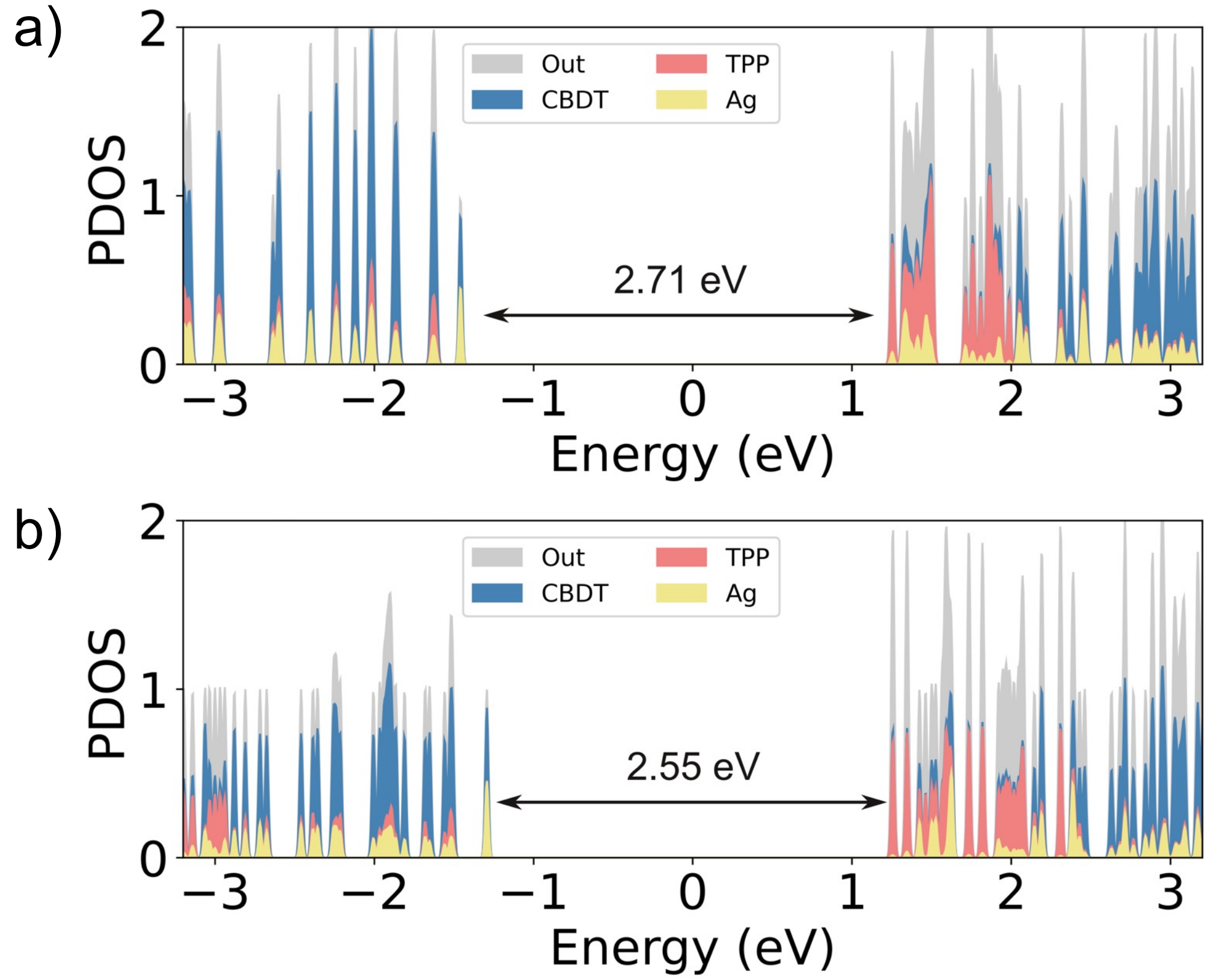

Figure S11. Projected density of electronic states (PDOS) (a) $Ag_{14}T$ and (b) $Ag_{14}S$ to the specified atom groups. “Out” denotes weights from the spatial distribution of electrons that are not captured by the analysis. Values of the HOMO-LUMO energy gaps are shown as well.

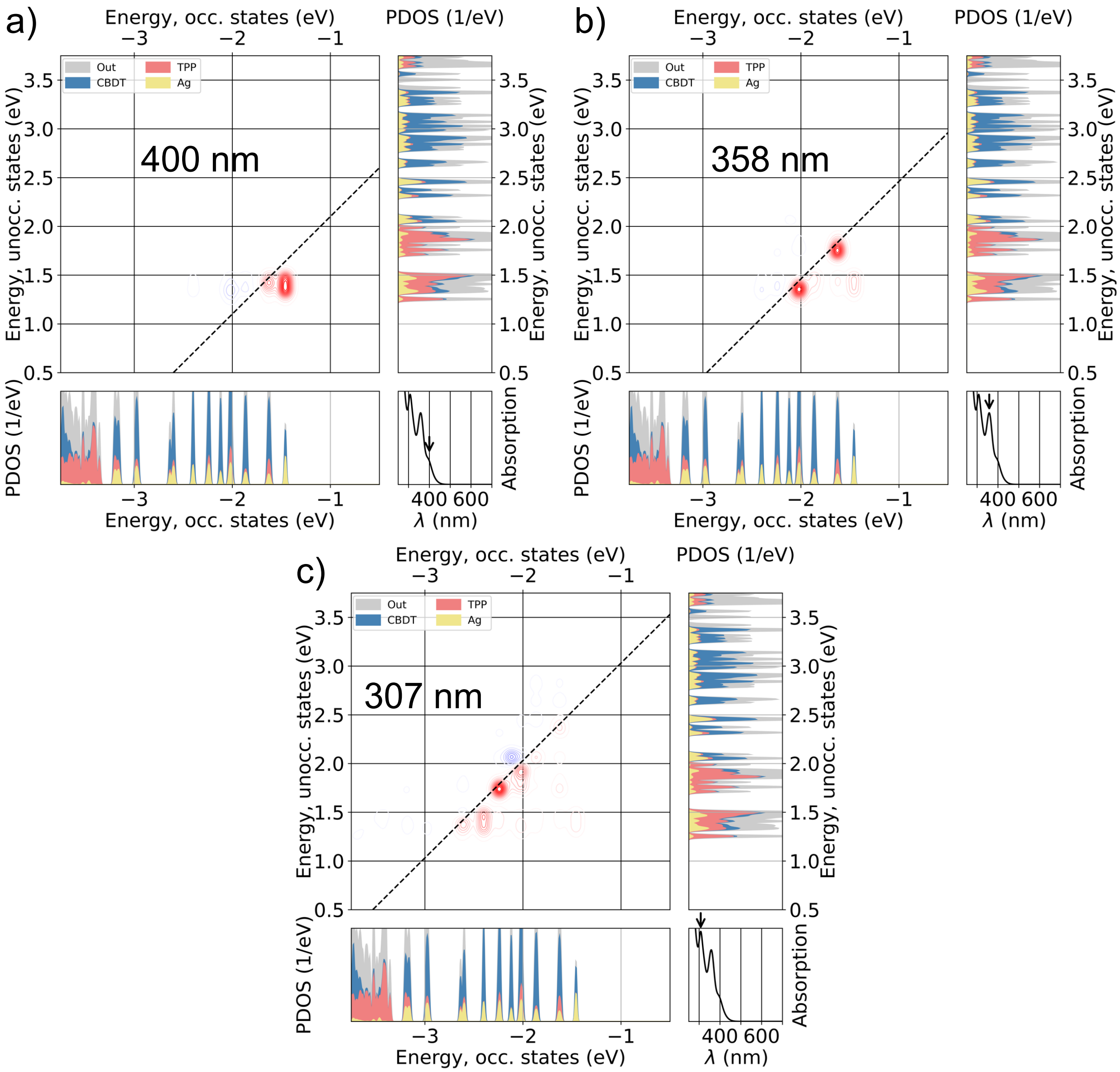

Figure S12. Dipole Transition Contribution Map (DTCM) analysis for the Ag_{14}T NC. In each collage of panels, the bottom right corner reproduces the calculated absorption spectrum and the arrow indicates the analyzed peak whose position is also given in nm. The correlation plot shows the contributions to the total transition dipole moment of the given peak by single-electron transitions from occupied Kohn-Sham orbitals (bottom left panel) to the empty orbitals (right vertical panel). The Kohn−Sham orbitals are projected onto the natural components of the system as in Figure S6.

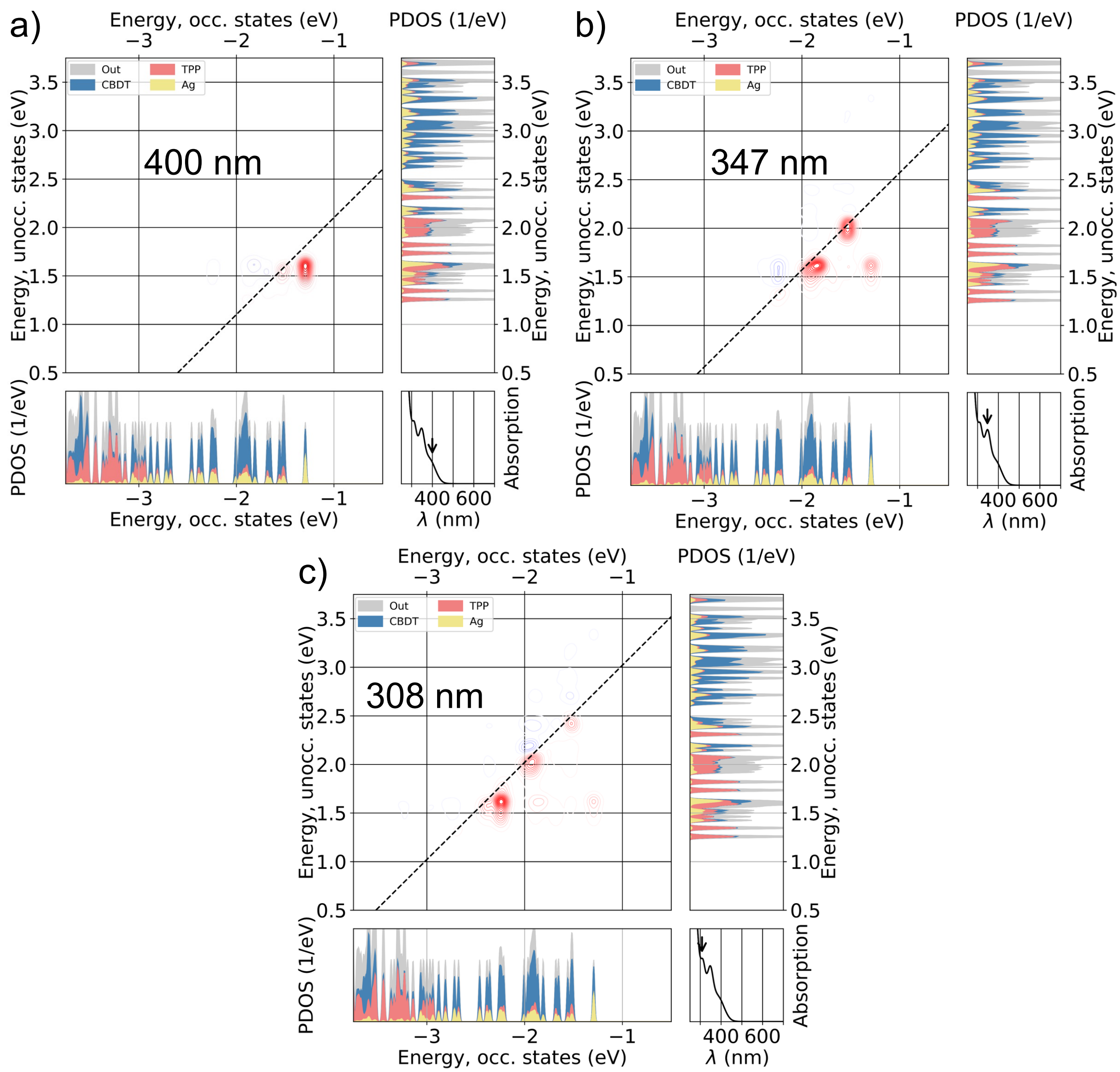

Figure S13. Dipole Transition Contribution Map (DTCM) analysis for the $Ag_{14}S$ NC. In each collage of panels, the bottom right corner reproduces the calculated absorption spectrum and the arrow indicates the analyzed peak whose position is also given in nm. The correlation plot shows the contributions to the total transition dipole moment of the given peak by single electron transitions from occupied Kohn-Sham orbitals (bottom left panel) to the empty orbitals (right vertical panel). The Kohn−Sham orbitals are projected onto the natural components of the system as in Figure S6.

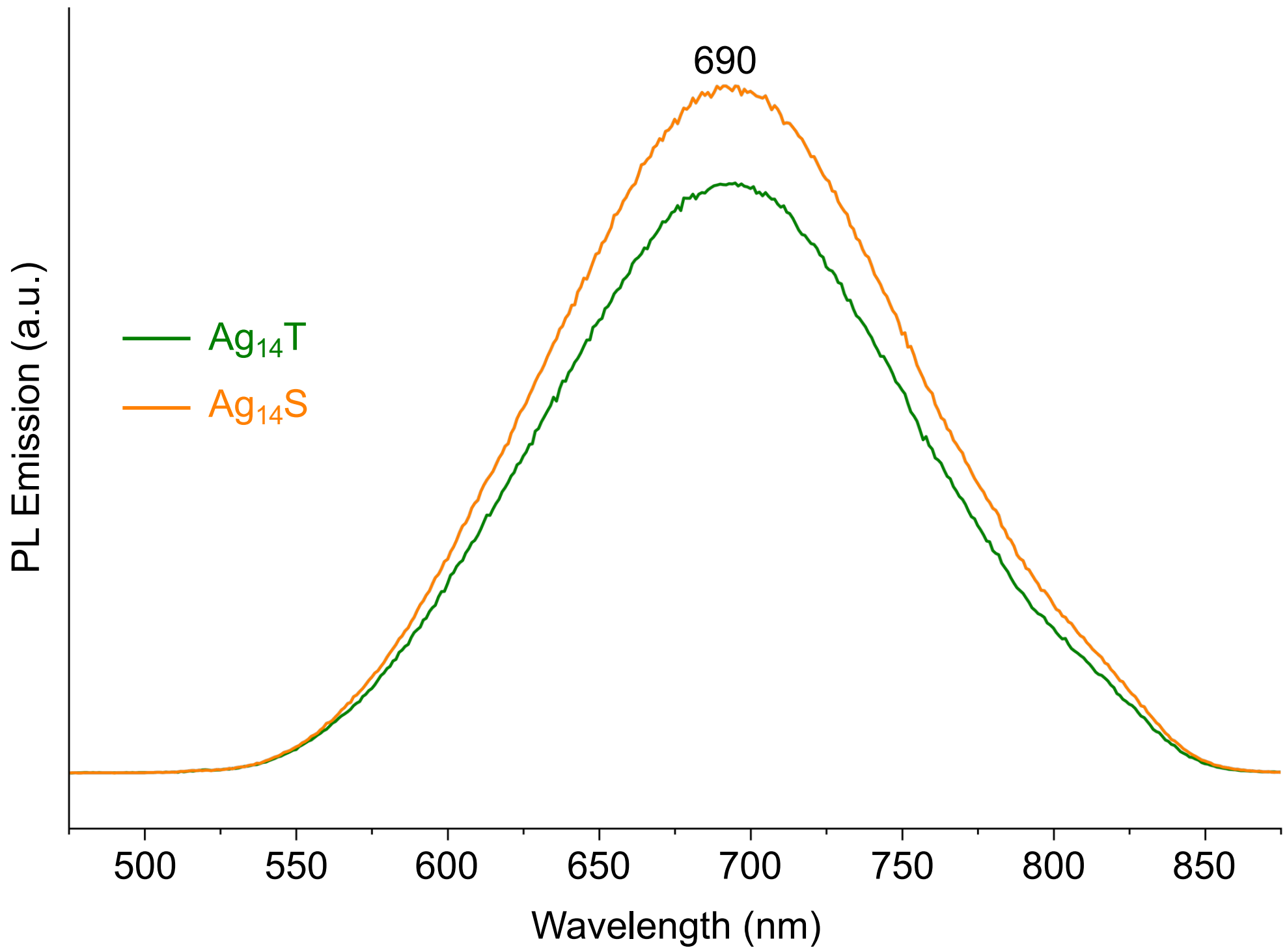

Figure S14. PL spectra of $Ag_{14}T$ and $Ag_{14}S$ in DMF and DMF:MeOH (1:1, v:v) solution, respectively showing a broad emission maxima at 690 nm upon 450 nm excitation.

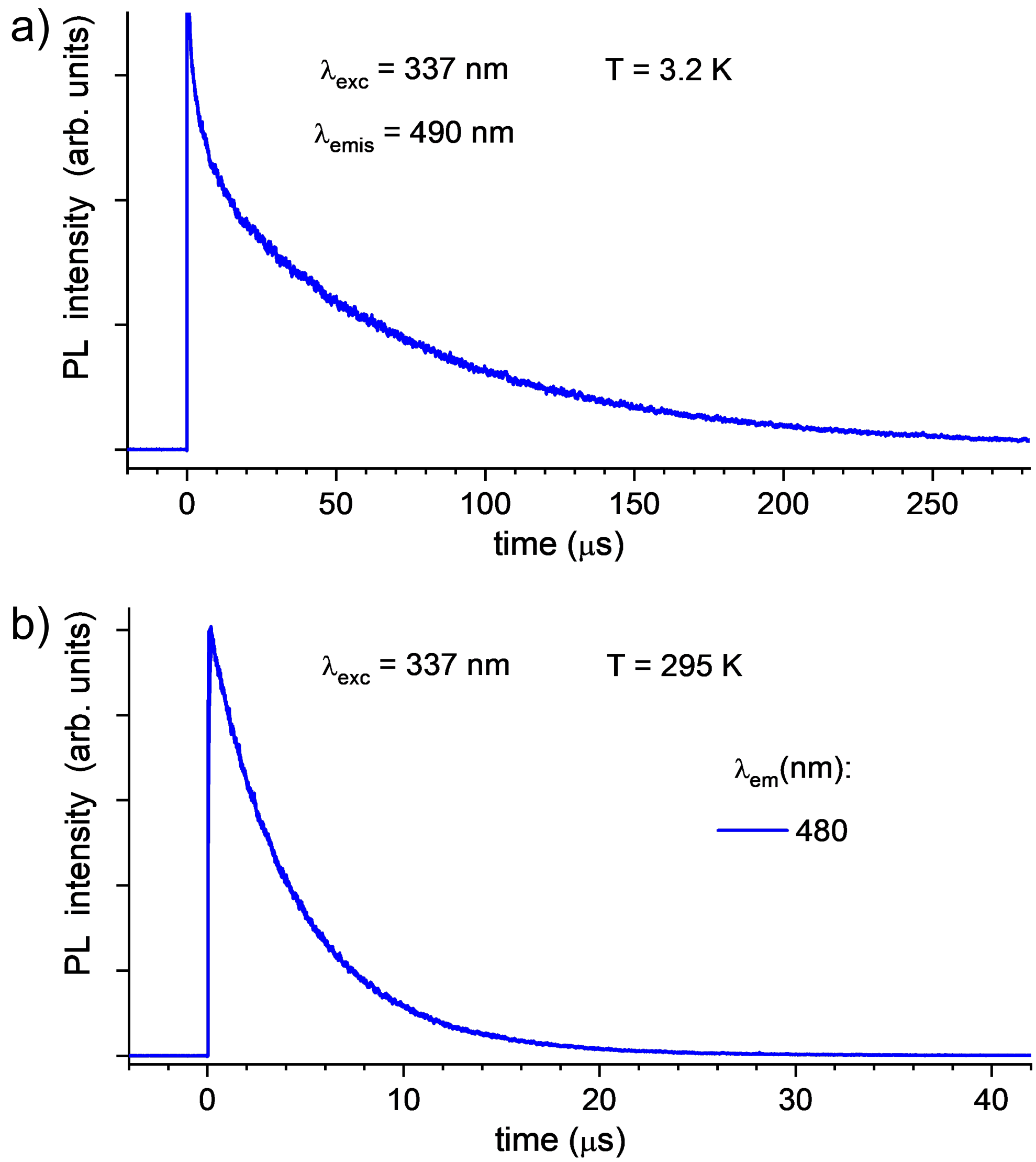

Figure S15. Emission decay of solid crystalline $Ag_{14}T$ NC at 3.2 and 295 K. The emission was excited with ns-laser pulses at 337 nm. The decay traces at $\lambda_{emi.}$ = 490 nm and 480 nm for 3.2 K and 295 K respectively, can be fit with monoexponential curves with the lifetime of 72 μs at T = 3.2 K and 4.7 μs at T = 295 K.

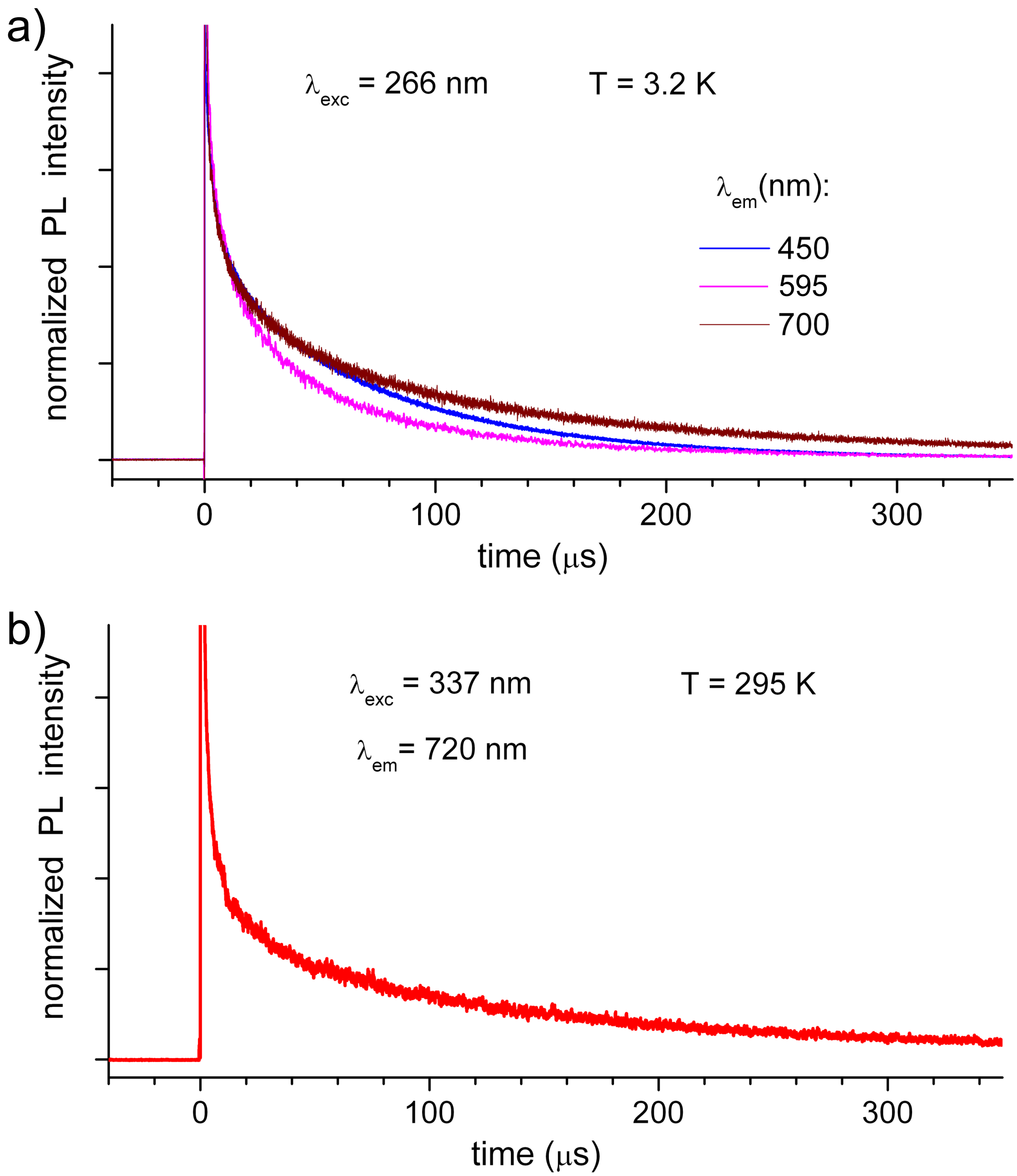

Figure S16. Emission decay of solid $Ag_{14}S$ at T = 3.2 and 295 K. The emission was excited with ns-laser pulses at 266 or 337 nm. The decay traces can be fit with biexponential curves with the following average lifetimes: $\lambda_{emi.}$ = 450, 595 and 700 nm at 3.2 K: 43, 26 and 66 μs, respectively; $\lambda_{emi.}$ = 720 nm at 295 K: 63 μs.

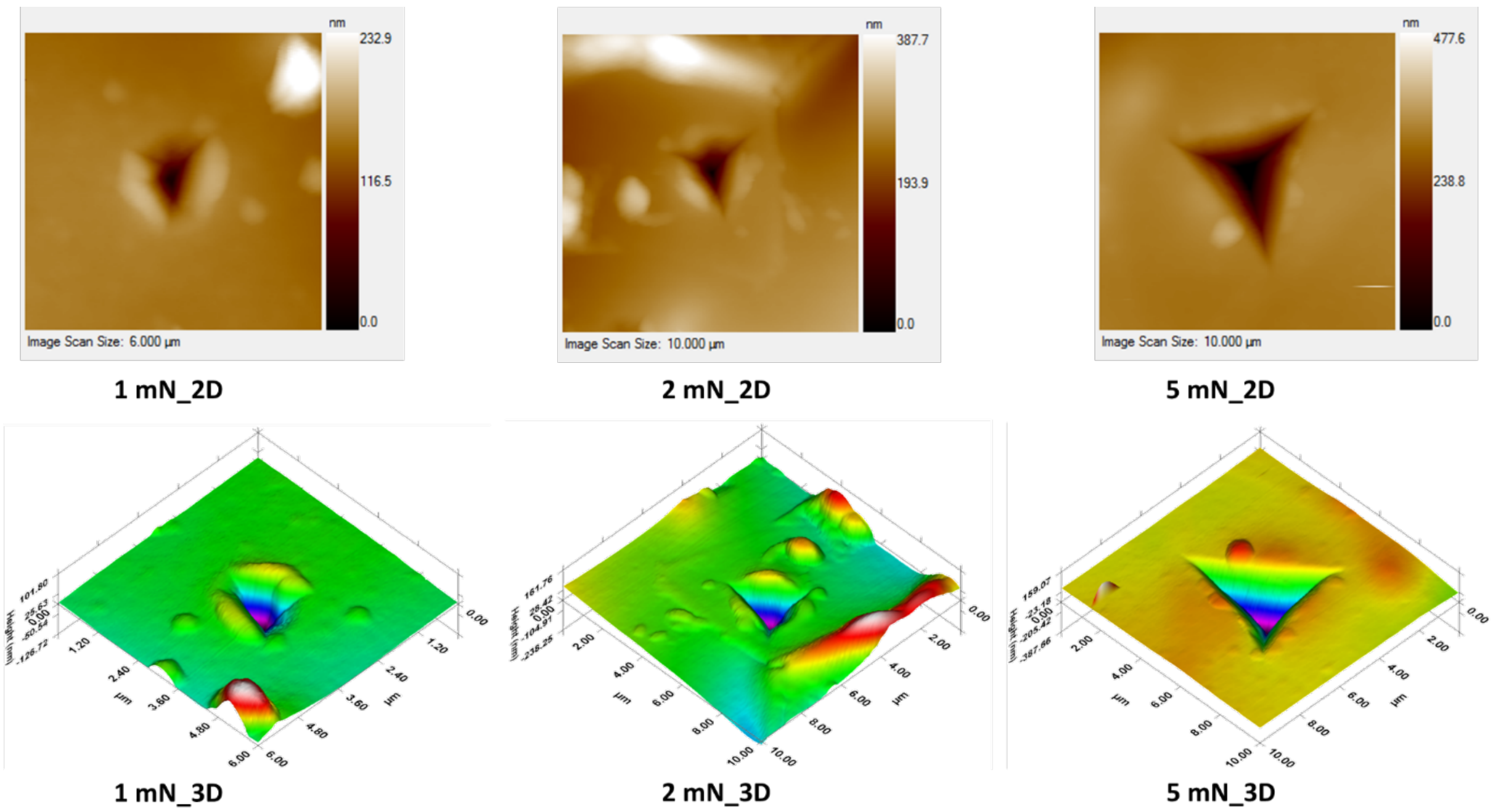

Figure S17. 2D & 3D indent impression images of $Ag_{14}T$ at different loads.

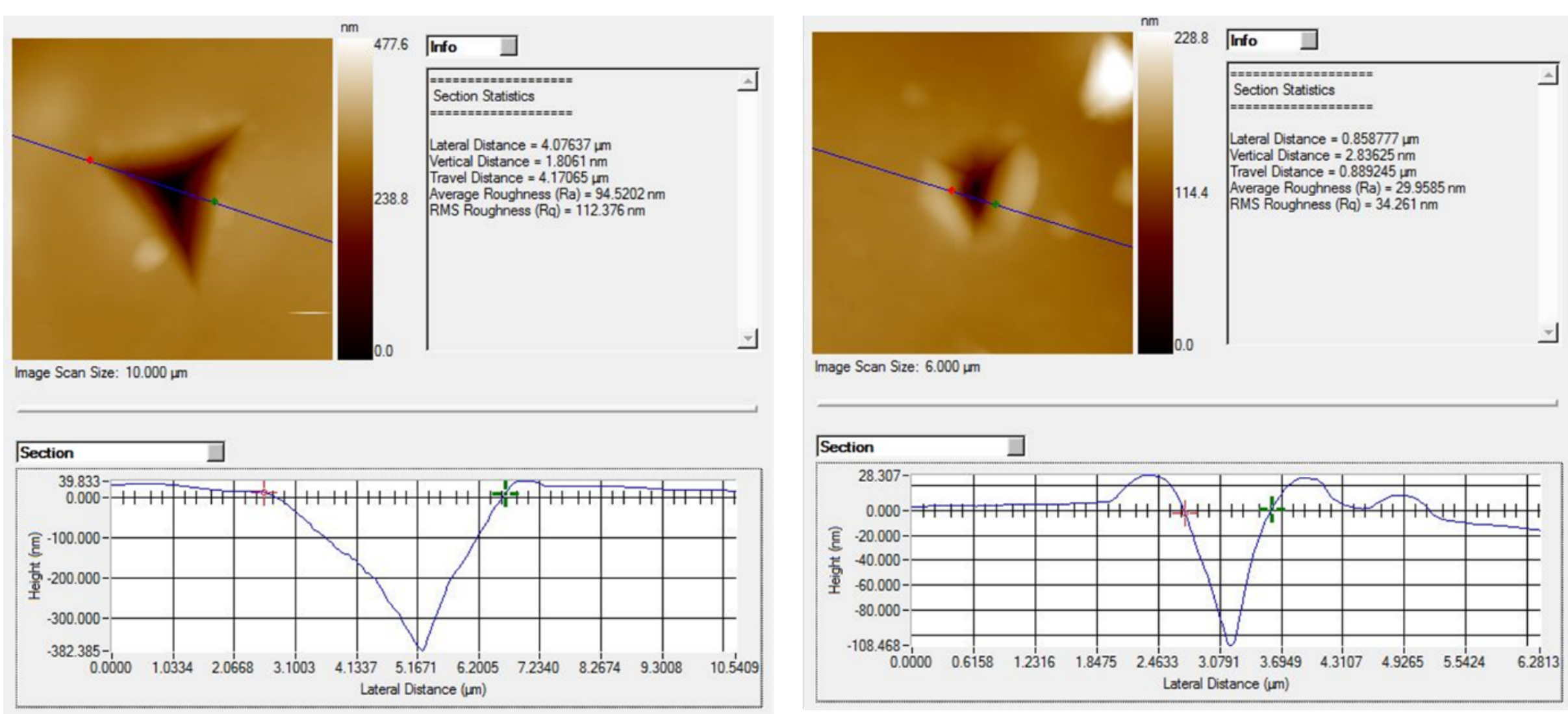

Height profile_5 mN

Height profile_1 mN

Figure S18. Height profile of $Ag_{14}T$ at different loads.

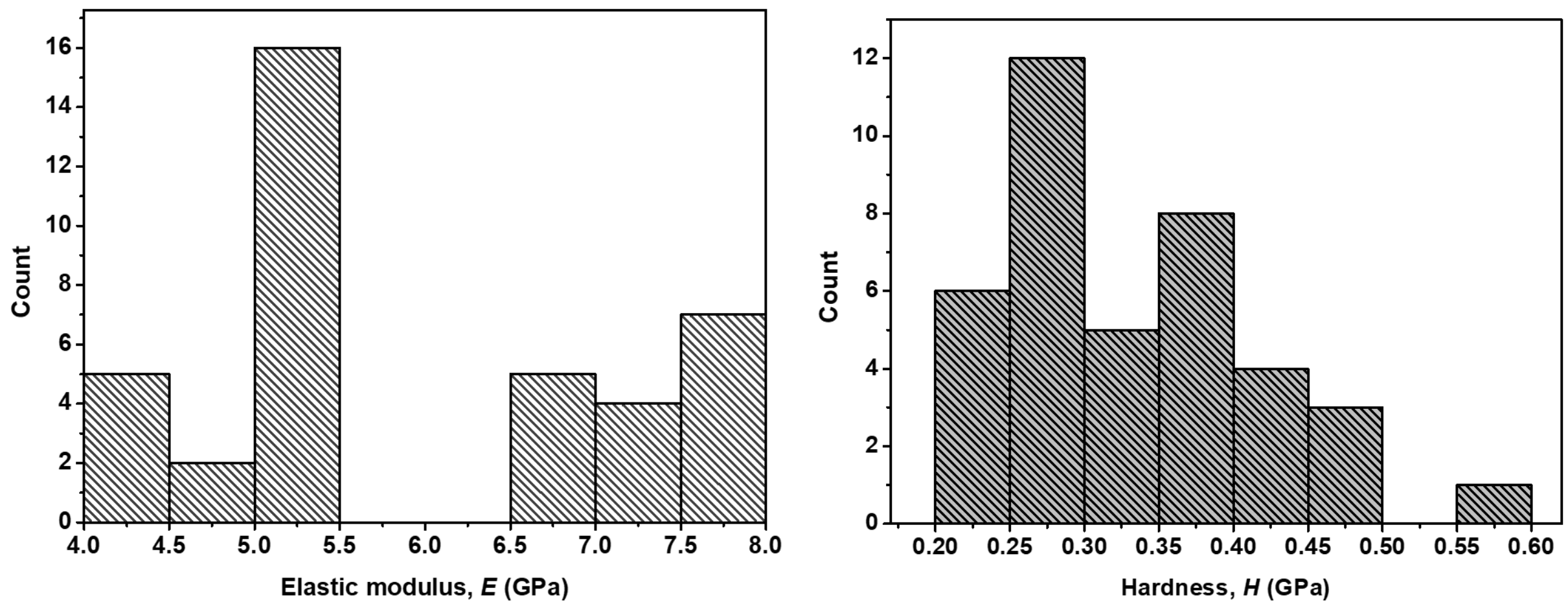

Figure S19. Histogram plots of elastic modulus and hardness of $Ag_{14}T$, combining the *E* and *H* values together obtained from different loads (1mN, 2 mN & 5 mN).

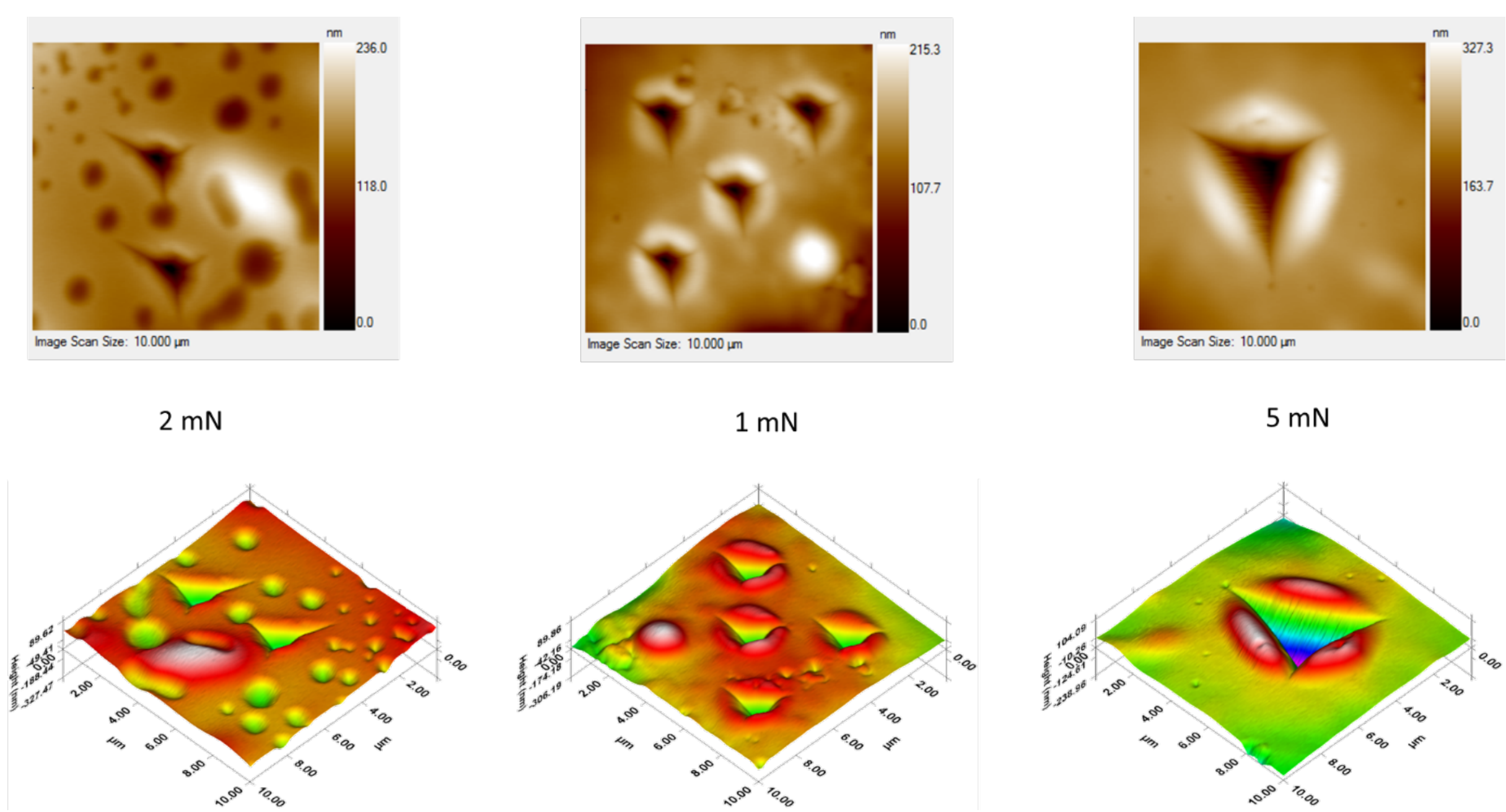

Figure S20. 2D & 3D indent impression images of $Ag_{14}S$ at different loads.

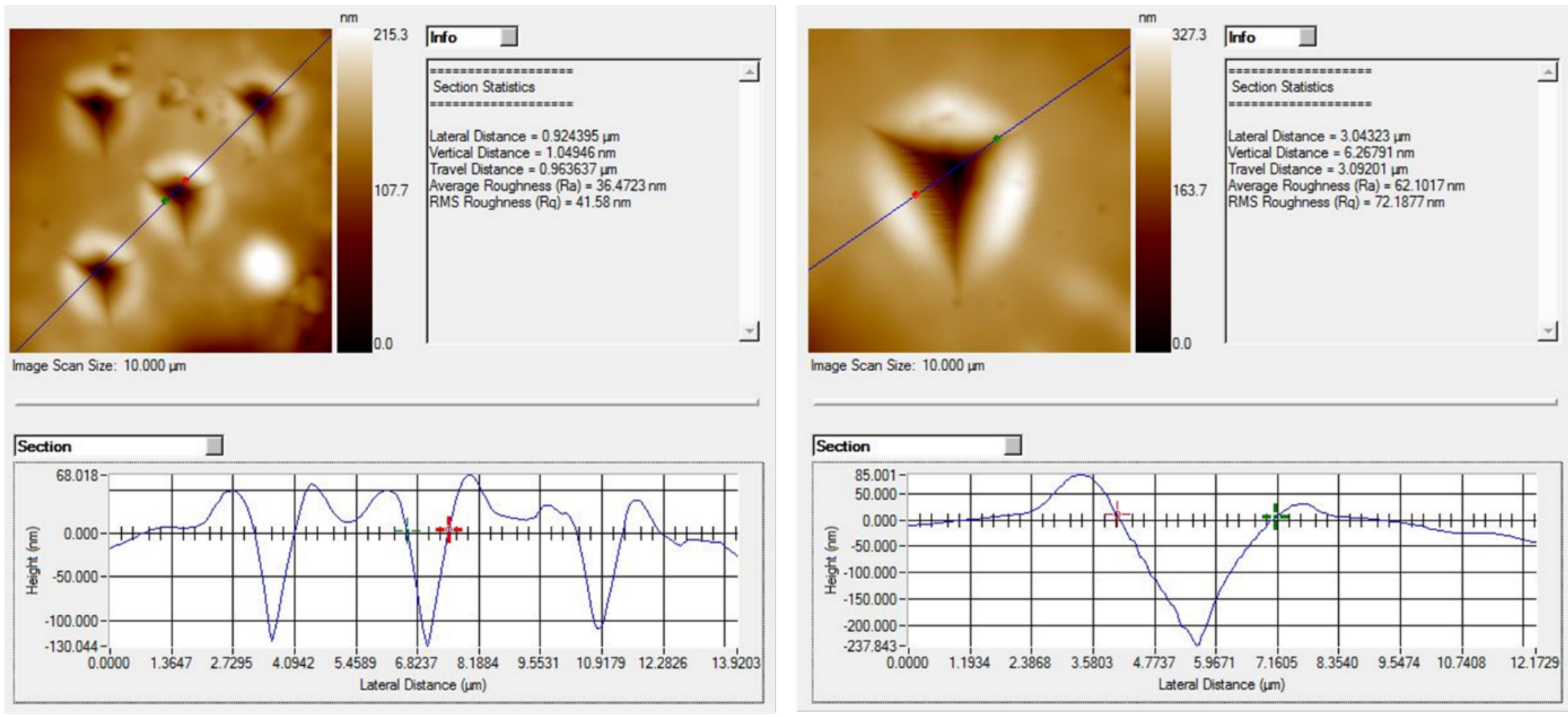

1 mN **5 mN**

Figure S21. Height profile of the $Ag_{14}S$ at different loads.

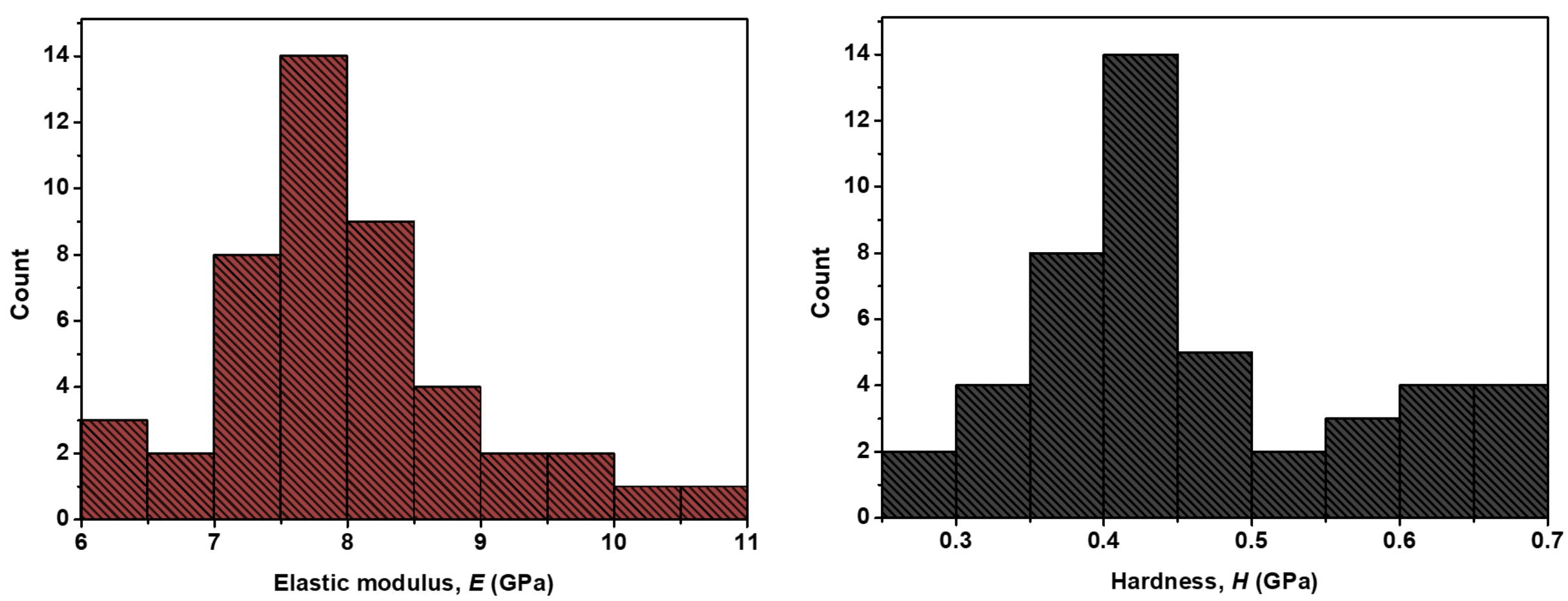

Figure S22. Histogram plots of elastic modulus and hardness of $Ag_{14}S$, combining the *E* and *H* values together obtained from different loads (1mN, 2 mN & 5 mN).

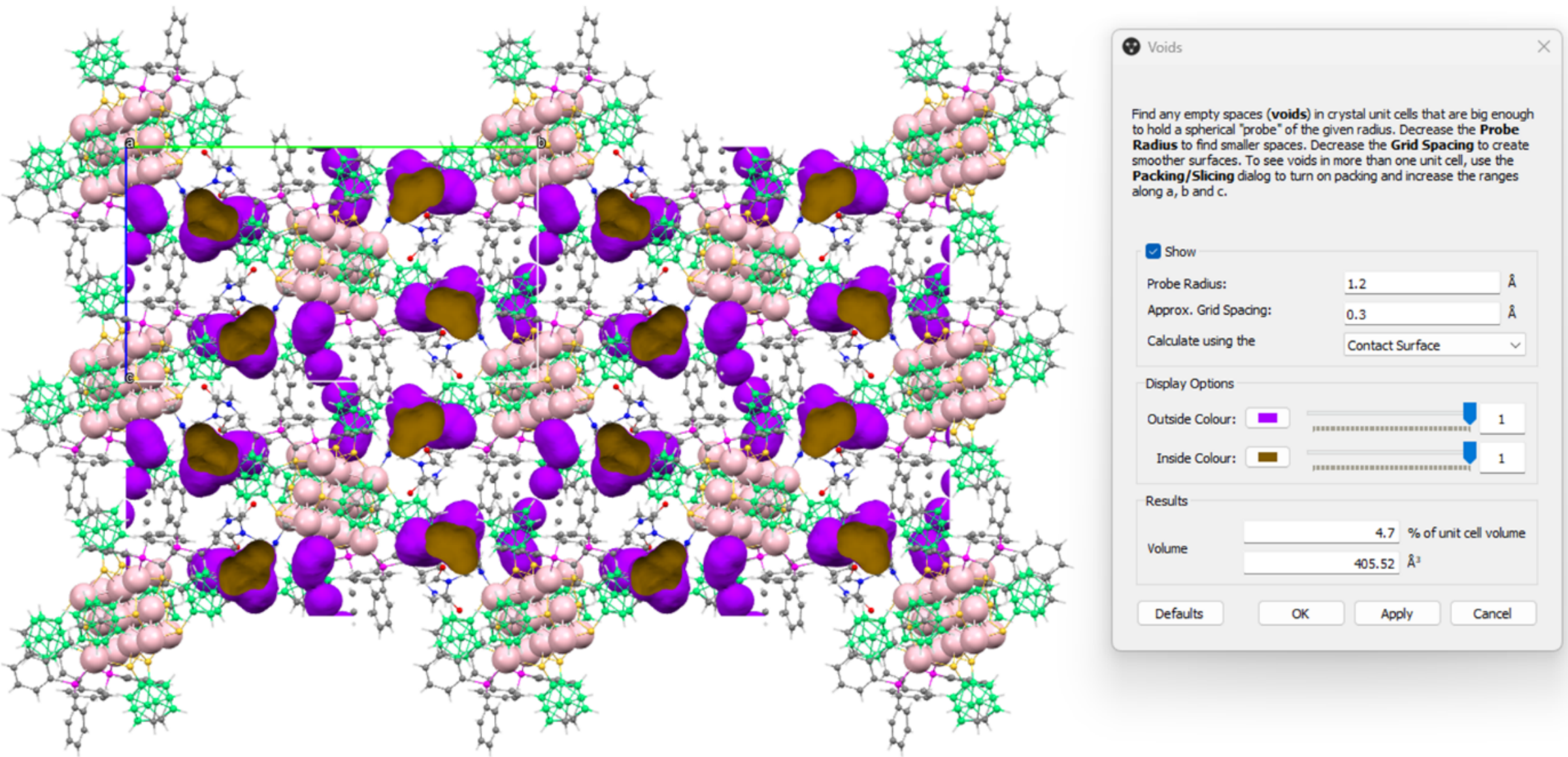

Figure S23. Output result of void calculation of $Ag_{14}S$ using CIF file in the Mercury software.

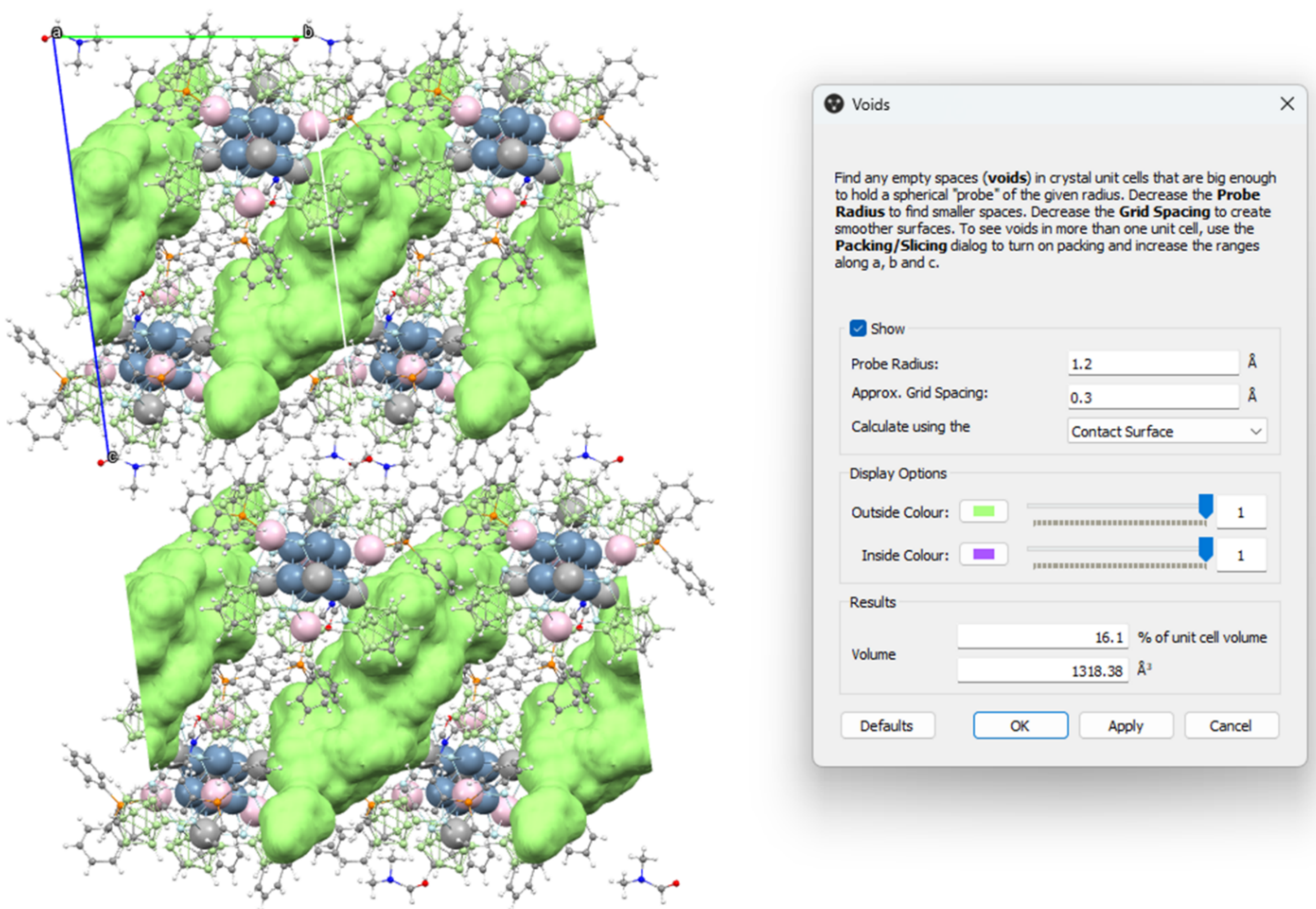

Figure S24. Output result of void calculation of $Ag_{14}T$ using CIF file in the Mercury software.

Table S1. Nanomechanical properties of isomeric Ag_{14} nanocluster crystals (σ represents standard deviation).

Systems		1mN	2mN	5mN
Ag_{14}S (Orange)	Elastic modulus (E)	8.48 (σ ≈ 0.9)	7.9 (σ ≈ 0.77)	7.26 (σ ≈ 0.78)
	Hardness (H)	534 (σ ≈ 110)	438 (σ ≈ 85)	373 (σ ≈ 63)
Ag_{14}T (Green)	Elastic modulus (E)	6.05 (σ ≈ 1.23)	5.6 (σ ≈ 1.37)	6.19 (σ ≈ 0.96)
	Hardness (H)	340 (σ ≈ 79)	323 (σ ≈ 109)	344 (σ ≈ 49)

Table S2. Crystallographic information of Ag_{14}T, and Ag_{14}S cluster crystals.

	Ag_{14}T	Ag_{14}S
Chemical formula	$C_{90} H_{134} Ag_{14} B_{60} P_4 S_{12} N_2 O_2$	$C_{101} H_{155} Ag_{14} B_{60} P_4 S_{12} N_5 O_7$
Formula weight	3943.36	4218.67
Temperature	298(2) K	296(2) K
Crystal system	Triclinic	Monoclinic
Space group	$P\bar{1}$	$P21/c$
Crystal Size (mm^3)	0.326 × 0.225 × 0.105	0.187 × 0.047 × 0.029
a (Å)	17.9307(16)	17.1781(6)
b (Å)	18.1195(16)	29.8349(11)
c (Å)	28.602(2)	18.2617(6)
α (°)	78.365(3)	90
β (°)	78.304(3)	111.5700(10)

γ (°)	65.005(3)	90
V (Å)^3	8176.8(13)	8703.8(5)
Z	2	2
Density calc. (mg m^{-3})	1.602	1.610
Absorption coefficient	1.866 mm^{-1}	1.761 mm^{-1}
F(000)	3828	4124
Theta range for data collection	2.476 to 26.000°.	2.460 to 25.500°.
Index ranges	-22<=h<=22,<br>-22<=k<=22,<br>-35<=l<=35	-20<=h<=20,<br>-36<=k<=36,<br>-21<=l<=22
Reflections collected	398520	187812
Independent reflections	32105 [R(int) = 0.0469]	16170 [R(int) = 0.0847]
Completeness to theta	99.8 %	99.8 %
Max. and min. transmission	0.7461 and 0.6344	0.7906 and 0.4167
Data / restraints / parameters	32105 / 245 / 1704	16170 / 405 / 1042
Goodness-of-fit on F^2	1.174	1.107
Final R indices [I>2sigma(I)]	R1 = 0.0477, wR2 = 0.1001	R1 = 0.0494, wR2 = 0.1080
R indices (all data)	R1 = 0.0697, wR2 = 0.1243	R1 = 0.0718, wR2 = 0.1191
Largest diff. peak and hole	1.938 and -1.624 e.Å^{-3}	1.652 and -0.804 e.Å^{-3}
CCDC No.	2533217	2533218

Table S3. Atomic coordinates (x 10^4) and equivalent isotropic displacement parameters ($Å^2$x 10^3) for $Ag_{14}T$. U(eq) is defined as one third of the trace of the orthogonalized U^{ij} tensor.

	x	y	z	U(eq)
Ag(1)	5152(1)	2515(1)	7851(1)	54(1)
Ag(2)	7766(1)	3971(1)	8250(1)	52(1)
Ag(3)	8339(1)	2102(1)	7986(1)	35(1)
Ag(4)	7116(1)	1837(1)	8785(1)	47(1)
Ag(5)	9211(1)	99(1)	7899(1)	52(1)
Ag(6)	8447(1)	1711(1)	7070(1)	35(1)
Ag(7)	7342(1)	1005(1)	6884(1)	46(1)
Ag(8)	9633(1)	2336(1)	7238(1)	46(1)
Ag(9)	7857(1)	3356(1)	7219(1)	35(1)
Ag(10)	6104(1)	4468(1)	7171(1)	49(1)
Ag(11)	6771(1)	2734(1)	7043(1)	36(1)
Ag(12)	8049(1)	3050(1)	6096(1)	53(1)
Ag(13)	6658(1)	3130(1)	7961(1)	36(1)
Ag(14)	7246(1)	1484(1)	7813(1)	36(1)
B(10)	5587(5)	4046(5)	8887(3)	45(2)
B(8)	4873(5)	4337(5)	8443(3)	41(2)
B(19)	7214(5)	-325(4)	8089(3)	39(2)
B(18)	6364(5)	330(5)	8479(3)	40(2)
B(30)	9703(5)	2049(5)	8593(3)	41(2)
B(26)	9413(5)	1180(4)	8866(3)	39(2)
B(38)	8743(5)	4507(5)	6561(3)	42(2)
B(40)	7901(5)	5157(5)	6973(3)	41(2)
B(46)	10195(5)	467(4)	6576(3)	39(2)
B(50)	9458(5)	741(4)	6149(3)	38(2)
B(60)	5366(5)	2801(5)	6472(3)	43(2)
B(54)	5653(5)	3647(5)	6181(3)	45(2)
C(13)	11241(5)	-1459(5)	8240(3)	61(2)
C(14)	11618(7)	-1947(7)	8633(5)	113(5)
C(15)	12400(8)	-2019(9)	8681(7)	151(7)
C(16)	12772(8)	-1569(9)	8367(7)	136(6)

C(17)	12385(7)	-1066(8)	7991(5)	108(4)
C(18)	11611(6)	-987(6)	7930(4)	76(3)
C(19)	9895(4)	-1947(5)	8642(3)	50(2)
C(20)	9389(5)	-1544(6)	9021(3)	62(2)
C(21)	9092(6)	-1967(8)	9422(3)	80(3)
C(22)	9308(7)	-2795(8)	9439(4)	88(3)
C(23)	9803(7)	-3199(7)	9062(4)	80(3)
C(24)	10094(6)	-2782(5)	8676(3)	66(2)
C(25)	10519(5)	-2006(4)	7643(3)	51(2)
C(26)	11333(6)	-2530(6)	7490(4)	80(3)
C(27)	11483(9)	-2977(7)	7120(5)	106(5)
C(28)	10853(11)	-2945(7)	6907(4)	109(5)
C(29)	10052(8)	-2434(6)	7053(3)	82(3)
C(30)	9887(6)	-1971(5)	7419(3)	58(2)
C(31)	3226(5)	2602(6)	8664(3)	69(2)
C(32)	3729(6)	2211(7)	9024(3)	76(3)
C(33)	3409(8)	2271(10)	9504(4)	115(5)
C(34)	2593(9)	2729(11)	9624(4)	144(7)
C(35)	2077(8)	3127(12)	9270(5)	165(8)
C(36)	2394(7)	3061(9)	8792(4)	118(5)
C(37)	3700(5)	1544(5)	7938(3)	62(2)
C(38)	3261(6)	1124(7)	8250(4)	88(3)
C(39)	3339(8)	378(8)	8142(6)	109(4)
C(40)	3818(7)	53(7)	7743(6)	108(5)
C(41)	4231(6)	461(6)	7440(5)	85(3)
C(42)	4182(5)	1204(6)	7535(4)	67(2)
C(43)	2940(5)	3298(5)	7689(3)	59(2)
C(44)	2339(6)	3159(6)	7519(4)	81(3)
C(45)	1785(7)	3788(9)	7243(5)	111(4)
C(46)	1833(8)	4540(9)	7118(5)	118(5)
C(47)	2415(9)	4671(8)	7293(5)	117(5)
C(48)	2972(6)	4064(6)	7568(4)	78(3)
C(49)	7830(5)	3182(5)	4823(3)	53(2)
C(50)	7706(5)	2458(5)	4973(3)	61(2)
C(51)	7300(5)	2213(7)	4710(4)	73(3)
C(52)	7026(6)	2707(8)	4296(4)	90(4)

C(53)	7150(7)	3407(8)	4131(4)	92(4)
C(54)	7548(6)	3658(6)	4398(3)	72(3)
C(55)	8034(5)	4571(5)	5014(3)	55(2)
C(56)	7298(5)	5085(5)	5239(3)	56(2)
C(57)	7045(6)	5929(5)	5124(3)	68(2)
C(58)	7511(7)	6257(6)	4791(4)	78(3)
C(59)	8233(8)	5750(6)	4564(4)	102(4)
C(60)	8515(7)	4912(6)	4674(4)	86(3)
C(61)	9436(5)	3024(4)	4937(3)	51(2)
C(62)	9703(6)	2630(5)	4532(3)	63(2)
C(63)	10534(6)	2263(6)	4367(4)	74(3)
C(64)	11121(7)	2276(6)	4597(4)	80(3)
C(65)	10869(6)	2677(7)	4994(4)	82(3)
C(66)	10036(6)	3046(6)	5169(3)	65(2)
C(67)	6931(5)	5928(4)	8770(3)	51(2)
C(68)	6508(5)	6295(5)	8376(3)	63(2)
C(69)	5749(6)	6981(6)	8417(5)	83(3)
C(70)	5441(6)	7285(6)	8855(5)	88(4)
C(71)	5864(8)	6933(6)	9237(4)	88(3)
C(72)	6611(6)	6262(5)	9195(3)	71(3)
C(73)	8695(5)	5334(5)	8432(3)	48(2)
C(74)	9477(5)	4742(5)	8322(3)	64(2)
C(75)	10137(6)	4946(6)	8114(4)	78(3)
C(76)	10007(6)	5756(7)	7982(4)	78(3)
C(77)	9230(7)	6353(6)	8081(4)	79(3)
C(78)	8576(6)	6150(5)	8307(3)	63(2)
C(79)	8096(5)	4607(5)	9323(3)	53(2)
C(80)	8580(7)	4829(7)	9536(4)	86(3)
C(81)	8748(8)	4473(9)	10006(4)	109(4)
C(82)	8461(7)	3903(8)	10253(4)	94(4)
C(83)	7980(6)	3700(6)	10046(3)	74(3)
C(84)	7787(5)	4036(5)	9583(3)	55(2)
S(1)	6695(1)	3369(1)	8812(1)	39(1)
S(2)	5141(1)	3984(1)	7843(1)	41(1)
S(3)	6096(1)	1434(1)	8498(1)	40(1)
S(4)	7931(1)	6(1)	7617(1)	39(1)

S(5)	8663(1)	900(1)	8666(1)	40(1)
S(6)	9276(1)	2794(1)	8069(1)	37(1)
S(7)	7186(1)	4819(1)	7437(1)	41(1)
S(8)	9007(1)	3411(1)	6539(1)	41(1)
S(9)	9962(1)	830(1)	7174(1)	38(1)
S(10)	8348(1)	1450(1)	6241(1)	39(1)
S(11)	6426(1)	3928(1)	6357(1)	42(1)
S(12)	5818(1)	2046(1)	6985(1)	39(1)
P(1)	10238(1)	-1356(1)	8118(1)	47(1)
P(2)	3713(1)	2507(1)	8044(1)	53(1)
P(3)	8352(1)	3464(1)	5198(1)	49(1)
P(4)	7865(1)	4989(1)	8705(1)	46(1)
B(1)	5165(6)	5087(5)	8581(3)	52(2)
B(2)	4104(6)	5352(6)	8515(4)	60(2)
B(3)	3874(5)	4481(7)	8768(4)	62(3)
B(4)	4768(5)	3684(6)	8999(3)	52(2)
B(5)	5237(6)	4924(6)	9203(4)	64(3)
B(6)	4991(6)	4046(7)	9466(3)	66(3)
B(7)	3946(7)	4303(9)	9387(4)	79(3)
C(1)	3645(6)	5268(7)	9081(3)	82(3)
B(9)	4345(7)	5707(7)	8973(5)	77(3)
C(2)	4265(6)	5030(7)	9479(4)	86(3)
B(11)	6164(5)	-58(5)	8007(3)	48(2)
B(12)	7243(5)	-473(5)	8726(3)	47(2)
B(13)	7584(5)	-1346(5)	8415(3)	51(2)
B(14)	6908(6)	-1090(5)	7973(4)	54(2)
B(15)	6238(6)	-309(6)	9026(4)	59(2)
B(16)	5559(5)	-44(5)	8581(4)	55(2)
B(17)	5893(6)	-892(6)	8275(4)	65(3)
C(3)	5993(5)	-1019(5)	8866(4)	67(2)
C(4)	6752(5)	-1606(5)	8516(3)	62(2)
B(20)	6982(6)	-1324(6)	8986(4)	61(2)
B(21)	9114(5)	2048(5)	9182(3)	45(2)
B(22)	9497(6)	1033(6)	9494(3)	56(2)
B(23)	10308(6)	389(5)	9099(4)	59(2)
B(24)	10439(5)	1013(5)	8552(4)	54(2)

B(25)	11119(7)	745(7)	8978(4)	74(3)
C(5)	10504(6)	812(6)	9516(3)	71(3)
B(27)	9798(7)	1792(7)	9615(4)	67(3)
B(28)	10748(6)	1752(6)	8670(4)	57(2)
B(29)	9929(6)	2396(6)	9071(3)	52(2)
C(6)	10750(6)	1584(6)	9277(3)	72(3)
B(31)	8977(6)	4894(6)	7022(3)	50(2)
B(32)	9536(6)	4898(6)	6438(3)	56(2)
B(33)	8821(6)	5170(6)	6013(3)	55(2)
B(34)	7827(6)	5328(5)	6347(3)	47(2)
B(35)	7528(6)	6171(5)	6662(3)	53(2)
B(36)	8236(6)	5904(5)	7088(3)	55(2)
B(37)	9240(6)	5726(6)	6760(4)	59(2)
C(7)	9086(6)	5868(5)	6173(3)	63(2)
B(39)	8080(7)	6174(6)	6083(4)	61(2)
C(8)	8344(6)	6445(5)	6534(3)	63(2)
B(41)	10288(5)	1097(5)	6016(3)	47(2)
B(42)	11188(6)	299(5)	6232(3)	53(2)
B(43)	10930(5)	-554(5)	6505(3)	50(2)
B(44)	9874(5)	-285(4)	6456(3)	43(2)
B(45)	10669(6)	-907(5)	6057(3)	58(2)
C(10)	11389(5)	-489(5)	5925(3)	59(2)
B(47)	11083(6)	478(6)	5614(3)	61(2)
B(48)	10031(6)	737(5)	5564(3)	51(2)
B(49)	9773(6)	-121(5)	5837(3)	51(2)
C(9)	10743(5)	-244(5)	5549(3)	62(2)
B(51)	5898(6)	2784(6)	5872(3)	54(2)
B(52)	5524(6)	3794(6)	5556(3)	61(2)
B(53)	5169(7)	3062(8)	5473(4)	73(3)
C(11)	4490(6)	4027(7)	5568(3)	79(3)
B(55)	4739(6)	4443(6)	5963(4)	65(3)
B(56)	5062(6)	2456(7)	6023(3)	61(2)
B(57)	4649(5)	3842(6)	6522(3)	53(2)
B(58)	4284(5)	3113(6)	6427(4)	58(2)
B(59)	3916(7)	4110(7)	6120(4)	73(3)
C(12)	4235(6)	3280(6)	5832(3)	76(3)

N(1)	2084(7)	1881(7)	6635(4)	112(4)
O(1)	3027(9)	2155(12)	6066(6)	238(8)
C(85)	1883(9)	1489(11)	7118(6)	156(7)
C(86)	1455(11)	2327(10)	6352(7)	164(7)
C(87)	2829(12)	1823(15)	6466(7)	197(11)
N(2)	6640(20)	1010(20)	10134(13)	182(14)
O(2)	7889(13)	-357(16)	10044(8)	142(10)
C(88)	6850(30)	1730(20)	10058(16)	220(20)
C(89)	7270(20)	265(19)	9923(15)	140(9)
C(90)	6200(30)	840(30)	10618(15)	241(19)
N(2')	6562(12)	640(14)	10105(7)	123(7)
O(2')	5252(16)	1383(17)	10139(9)	219(11)
C(88')	5906(13)	1403(16)	9932(8)	144(9)
C(89')	7428(13)	490(20)	9928(14)	218(17)
C(90')	6357(19)	-3(18)	10419(13)	219(15)

Table S4. Atomic coordinates (x 10^4) and equivalent isotropic displacement parameters ($Å^2$x 10^3)for $Ag_{14}S$. U(eq) is defined as one third of the trace of the orthogonalized U^{ij} tensor.

	x	y	z	U(eq)
Ag(1)	4919(1)	5770(1)	6843(1)	52(1)
Ag(2)	4500(1)	5134(1)	3835(1)	32(1)
Ag(3)	5477(1)	5606(1)	5263(1)	31(1)
Ag(4)	3971(1)	5206(1)	5114(1)	33(1)
Ag(5)	7382(1)	5257(1)	6548(1)	50(1)
Ag(6)	3944(1)	6026(1)	4209(1)	42(1)
Ag(7)	6086(1)	5597(1)	3979(1)	43(1)
P(1)	4708(1)	6240(1)	7913(1)	46(1)
P(2)	8874(1)	5402(1)	7472(1)	44(1)
C(1)	4554(5)	6832(2)	7666(4)	51(2)

C(2)	4036(6)	6945(3)	6924(5)	66(2)
C(3)	3917(8)	7388(4)	6693(6)	93(3)
C(4)	4311(8)	7724(3)	7200(7)	96(4)
C(5)	4844(8)	7621(3)	7920(6)	101(4)
C(6)	4965(7)	7177(3)	8160(5)	80(3)
C(7)	5502(5)	6239(3)	8903(5)	56(2)
C(8)	6257(8)	6039(6)	9078(9)	74(4)
C(9)	6816(11)	6019(6)	9862(10)	99(6)
C(10)	6624(12)	6175(7)	10486(12)	86(6)
C(11)	5840(10)	6373(5)	10314(9)	65(4)
C(12)	5273(11)	6398(5)	9534(8)	62(4)
C(8B)	6311(12)	6359(12)	8893(18)	103(9)
C(9B)	7024(17)	6433(11)	9584(17)	106(9)
C(10B)	6950(20)	6351(12)	10300(20)	101(9)
C(11B)	6190(20)	6200(14)	10322(18)	78(8)
C(12B)	5459(18)	6188(11)	9639(11)	73(8)
C(13)	3779(5)	6083(3)	8123(4)	51(2)
C(14)	3218(7)	6390(4)	8213(6)	88(3)
C(15)	2540(7)	6230(6)	8389(7)	107(4)
C(16)	2426(7)	5791(6)	8477(5)	99(4)
C(17)	2971(7)	5487(4)	8373(5)	90(4)
C(18)	3655(6)	5632(3)	8201(5)	67(2)
C(19)	9361(4)	5878(2)	7176(4)	46(2)
C(20)	9117(4)	5971(3)	6383(4)	48(2)
C(21)	9427(5)	6344(3)	6131(5)	58(2)
C(22)	9975(6)	6620(3)	6652(6)	71(3)
C(23)	10233(6)	6532(3)	7434(7)	81(3)
C(24)	9947(5)	6154(3)	7722(5)	66(2)
C(25)	9617(5)	4944(3)	7632(4)	50(2)
C(26)	10428(6)	4991(3)	7656(6)	78(3)
C(27)	10948(7)	4623(4)	7790(7)	100(4)
C(28)	10677(8)	4207(4)	7907(6)	91(3)
C(29)	9880(7)	4146(3)	7869(5)	77(3)
C(30)	9339(5)	4516(3)	7730(5)	60(2)
C(31)	8976(5)	5559(3)	8470(4)	56(2)
C(32)	9612(10)	5445(9)	9164(9)	73(5)

C(33)	9630(15)	5628(8)	9874(13)	94(6)
C(34)	9058(11)	5947(7)	9886(9)	103(5)
C(35)	8441(10)	6089(6)	9198(8)	87(5)
C(36)	8385(9)	5881(6)	8498(8)	74(4)
C(32B)	9779(18)	5500(30)	9030(20)	78(11)
C(33B)	9870(30)	5530(20)	9820(30)	89(10)
C(34B)	9210(20)	5657(17)	10048(19)	84(9)
C(35B)	8430(20)	5692(14)	9447(15)	81(8)
C(36B)	8292(15)	5625(14)	8658(17)	68(8)
B(3)	2538(4)	5823(2)	5361(4)	33(2)
B(8)	2094(4)	5261(2)	5129(4)	32(2)
B(15)	3399(5)	5578(3)	2147(4)	39(2)
B(20)	4349(5)	5375(3)	2050(4)	39(2)
B(24)	6451(5)	6460(2)	6236(4)	38(2)
B(29)	6008(5)	6639(2)	5205(4)	36(2)
B(1)	1693(5)	6207(3)	4951(5)	45(2)
B(2)	2065(5)	6077(3)	5967(5)	46(2)
C(37)	1029(5)	6098(3)	5413(5)	53(2)
B(4)	1357(5)	5701(3)	6121(5)	51(2)
B(5)	2303(5)	5503(3)	6085(4)	41(2)
B(6)	1707(5)	5712(3)	4428(5)	41(2)
B(7)	769(5)	5907(3)	4469(5)	53(2)
C(38)	635(4)	5603(3)	5206(5)	52(2)
B(9)	1357(5)	5199(3)	5603(5)	44(2)
B(10)	1008(5)	5327(3)	4594(5)	45(2)
B(11)	4156(6)	5965(3)	2027(5)	45(2)
B(12)	3075(6)	6054(3)	1511(5)	56(2)
B(13)	2572(6)	5523(3)	1202(5)	57(2)
B(14)	3361(6)	5098(3)	1524(5)	47(2)
C(39)	2883(6)	5851(3)	595(5)	68(2)
B(16)	3002(7)	5286(4)	535(5)	66(3)
B(17)	4084(7)	5197(3)	1045(5)	60(3)
B(18)	4569(7)	5732(3)	1351(5)	54(2)
B(19)	3785(7)	6145(3)	1041(5)	66(3)
C(40)	3720(6)	5670(3)	505(4)	64(2)
B(21)	5604(6)	6859(3)	5912(5)	47(2)

B(22)	6474(6)	6936(3)	6818(5)	53(2)
B(23)	7394(6)	6769(3)	6694(5)	53(2)
C(42)	7098(6)	7305(3)	6613(5)	68(2)
B(25)	6045(7)	7401(3)	6205(6)	64(3)
B(26)	7125(5)	6586(3)	5703(5)	44(2)
B(27)	5768(6)	7227(3)	5217(6)	49(2)
B(28)	6696(6)	7060(3)	5085(6)	54(2)
C(41)	6708(6)	7474(3)	5710(5)	65(2)
B(30)	7554(6)	7123(3)	5982(6)	62(3)
S(1)	3622(1)	5983(1)	5468(1)	35(1)
S(2)	3132(1)	5534(1)	3043(1)	38(1)
S(3)	5236(1)	5099(1)	2842(1)	36(1)
S(4)	7358(1)	5236(1)	5073(1)	35(1)
S(5)	5406(1)	6293(1)	4342(1)	35(1)
S(6)	6328(1)	5897(1)	6607(1)	35(1)
C(43)	7897(10)	6114(6)	3835(10)	185(9)
C(44)	6697(8)	6445(6)	2778(9)	170(8)
C(45)	8098(8)	6546(3)	2825(7)	91(3)
O(1)	7920(6)	6780(3)	2253(6)	130(3)
N(1)	7587(6)	6370(3)	3122(5)	92(3)
O(2)	-254(8)	6902(4)	4764(7)	175(5)
N(2)	-795(10)	7503(5)	4034(9)	167(7)
C(48)	-291(13)	7166(7)	4301(12)	178(8)
C(47)	-723(13)	7800(9)	3494(14)	320(20)
C(46)	-1461(15)	7536(9)	4310(19)	306(19)
O(4)	1534(10)	6660(7)	-355(9)	151(6)
C(52)	1141(19)	6811(13)	190(15)	209(16)
O(4')	1416(18)	7163(12)	639(16)	151(6)
C(52')	760(20)	6894(15)	720(20)	162(18)
N(3)	2411(10)	7165(5)	3531(9)	73(4)
O(3)	1123(17)	7304(9)	2926(14)	202(12)
C(49)	2281(17)	6741(7)	3179(13)	157(13)
C(50)	3194(12)	7244(8)	4148(15)	142(11)
C(51)	1758(14)	7459(8)	3419(17)	136(10)

References

1. Bruker (2021)– APEX4-SAINT Bruker AXS Inc., Madison, Wisconsin, USA.
2. SADABS-2016/2 - Bruker AXS area detector scaling and absorption Correction, Krause, L., Herbst-Irmer, R., Sheldrick G.M. & Stalke D., J. Appl. Cryst. 48 (2015) 3-10.
3. G.M. Sheldrick "SHELXT - Integrated space-group and crystal-structure determination", Acta Cryst., **2015**, A71, 3-8
4. G.M. Sheldrick , Crystal structure refinement with SHELXL", Acta Cryst., **2015**, C71,3-8
5. Farrugia, L. J. WinGX for Windows, *J. Appl. Crystallogr.* **2012**, *45*, 849-854.
6. A.L Spek (2009) Acta. Cryst. D65, 148-155
7. Mortensen, J. J.; Larsen, A. H.; Kuisma, M.; Ivanov, A. V.; Taghizadeh, A.; Peterson, A.; Haldar, A.; Dohn, A. O.; Schäfer, C.; Jónsson, E. Ö.; Hermes, E. D.; Nilsson, F. A.; Kastlunger, G.; Levi, G.; Jónsson, H.; Häkkinen, H.; Fojt, J.; Kangsabanik, J.; Sødequist, J.; Lehtomäki, J.; Heske, J.; Enkovaara, J.; Winther, K. T.; Dulak, M.; Melander, M. M.; Ovesen, M.; Louhivuori, M.; Walter, M.; Gjerding, M.; Lopez-Acevedo, O.; Erhart, P.; Warmbier, R.; Würdemann, R.; Kaappa, S.; Latini, S.; Boland, T. M.; Bligaard, T.; Skovhus, T.; Susi, T.; Maxson, T.; Rossi, T.; Chen, X.; Schmerwitz, Y. L. A.; Schiøtz, J.; Olsen, T.; Jacobsen, K. W.; Thygesen, K. S. GPAW: An Open Python Package for Electronic Structure Calculations. J. Chem. Phys. 2024, 160 (9), 092503.
8. Enkovaara J; Rostgaard C.; Mortensen J. J.; Chen J.; Dułak M.; Ferrighi L.; Gavnholt J.; Glinsvad C.; Haikola V.; Hansen H. A.; Kristoffersen H. H.; Kuisma M.; Larsen A. H.; Lehtovaara L.; Ljungberg M.; Lopez-Acevedo O.; Moses P. G.; Ojanen J; Olsen T.; Petzold V.; Romero N. A.; Stausholm-Møller J.; Strange M.; Tritsaris G. A.; Vanin M.; Walter M.; Hammer B.; Häkkinen H.; Madsen G. K. H.; Nieminen R. M.; Nørskov J. K.; Puska M.; Rantala T. T.; Schiøtz J.; Thygesen K. S.; Jacobsen K. W., Electronic structure calculations with GPAW: a real-space implementation of the projector augmented-wave method, J. Phys.: Condens. Matter 2010, 22, 253202.
9. Kuisma, M.; Ojanen, J.; Enkovaara, J.; Rantala, T. T. Kohn-Sham potential with discontinuity for band gap materials. Phys. Rev. B, 2010, 82, 115106.
10. Perdew J. P.; Burke K.; Ernzerhof M., Generalized Gradient Approximation Made Simple, Phys. Rev. Lett. 1996, 77, 3865-3868.
